# Phytochrome C and Low Temperature Promote the Protein Accumulation and Red-Light Signaling of Phytochrome D

**DOI:** 10.1093/pcp/pcae089

**Published:** 2024-08-09

**Authors:** Csaba Péter, Éva Ádám, Cornelia Klose, Gábor Grézal, Anita Hajdu, Gábor Steinbach, László Kozma-Bognár, Dániel Silhavy, Ferenc Nagy, András Viczián

**Affiliations:** Laboratory of Photo and Chronobiology, Institute of Plant Biology, Biological Research Centre, Hungarian Research Network (HUN-REN), Temesvari krt. 62, Szeged H-6726, Hungary; Doctoral School of Biology, Faculty of Sciences and Informatics, University of Szeged, Középfasor 52, Szeged H-6726, Hungary; Laboratory of Photo and Chronobiology, Institute of Plant Biology, Biological Research Centre, Hungarian Research Network (HUN-REN), Temesvari krt. 62, Szeged H-6726, Hungary; Institute of Biology II, University of Freiburg, Schänzlestr. 1, Freiburg 79104, Germany; Synthetic and Systems Biology Unit, Institute of Biochemistry, Biological Research Centre, Hungarian Research Network (HUN-REN), Temesvari krt. 62, Szeged H-6726, Hungary; HCEMM-BRC Metabolic Systems Biology Lab, Temesvari krt. 62, Szeged HU-6726, Hungary; Laboratory of Photo and Chronobiology, Institute of Plant Biology, Biological Research Centre, Hungarian Research Network (HUN-REN), Temesvari krt. 62, Szeged H-6726, Hungary; Cellular Imaging Laboratory, Biological Research Center, Hungarian Research Network (HUN-REN), Temesvari krt. 62, Szeged H-6726, Hungary; Laboratory of Photo and Chronobiology, Institute of Plant Biology, Biological Research Centre, Hungarian Research Network (HUN-REN), Temesvari krt. 62, Szeged H-6726, Hungary; Department of Genetics, Faculty of Sciences and Informatics, University of Szeged, Középfasor 52, Szeged H-6726, Hungary; Laboratory of Photo and Chronobiology, Institute of Plant Biology, Biological Research Centre, Hungarian Research Network (HUN-REN), Temesvari krt. 62, Szeged H-6726, Hungary; Laboratory of Photo and Chronobiology, Institute of Plant Biology, Biological Research Centre, Hungarian Research Network (HUN-REN), Temesvari krt. 62, Szeged H-6726, Hungary; Laboratory of Photo and Chronobiology, Institute of Plant Biology, Biological Research Centre, Hungarian Research Network (HUN-REN), Temesvari krt. 62, Szeged H-6726, Hungary

**Keywords:** *Arabidopsis*, Photomorphogenesis, phyD and phyC interaction, Phytochrome, Phytochrome D, Thermal reversion

## Abstract

Light affects almost every aspect of plant development. It is perceived by photoreceptors, among which phytochromes (PHY) are responsible for monitoring the red and far-red spectrum. *Arabidopsis thaliana* possesses five phytochrome genes (phyA–phyE). Whereas functions of phyA and phyB are extensively studied, our knowledge of other phytochromes is still rudimentary. To analyze phyD function, we expressed it at high levels in different phytochrome-deficient genetic backgrounds. Overexpressed phyD-YFP can govern effective light signaling but only at low temperatures and in cooperation with functional phyC. Under these conditions, phyD-YFP accumulates to high levels, and opposite to phyB, this pool is stable in light. By comparing the photoconvertible phyD-YFP and phyB levels and their signaling in continuous and pulsed irradiation, we showed that phyD-YFP is a less efficient photoreceptor than phyB. This conclusion is supported by the facts that only a part of the phyD-YFP pool is photoconvertible and that thermal reversion of phyD-YFP is faster than that of phyB. Our data suggest that the temperature-dependent function of phyD is based on the amount of phyD protein and not on its Pfr stability, as described for phyB. We also found that phyD-YFP and phyB-GFP are associated with strongly overlapping genomic locations and are able to mediate similar changes in gene expression; however, the efficiency of phyD-YFP is lower. Based on these data, we propose that under certain conditions, synergistic interaction of phyD and phyC can substitute phyB function in seedlings and in adult plants and thus increases the ability of plants to respond more flexibly to environmental changes.

## Introduction

Plants are sessile organisms and depend on light as an energy source utilized by photosynthesis; thus, it is essential to optimize their growth and development according to the surrounding light environment. In the dark, young seedlings undergo skotomorphogenesis developing long hypocotyls and small, closed, yellowish cotyledons, whereas under light irradiation photomorphogenesis takes place, resulting in seedlings having short hypocotyls and large, opened, green cotyledons. To achieve this, plants possess light-sensing photoreceptor molecules perceiving radiation on a wide spectrum, including ultraviolet (UV) B by the UVB-RESISTANCE LOCUS 8 (Rizzini et al. 2011), blue/UV-A by cryptochromes (Wang and Lin 2020), phototropins (Briggs and Christie 2002) and the family of Light-Oxygen-Voltage-sensing domain/F-box proteins (Demarsy and Fankhauser 2009). Phytochromes (PHY) are the sensors of red (R, ʎ_max_ ∼ 660 nm) and far-red (FR, ʎ_max_ ∼ 730 nm) light. *Arabidopsis thaliana*, a widely used model plant, has five phytochromes (phyA–phyE) (Quail 2002, Bae and Choi 2008). They appear as functional dimers of two ∼125 kDa monomers, each of them cradling a linear tetrapyrrol chromophore providing light sensitivity to the molecule. Phytochromes are synthesized in their inactive (Pr) form, and upon red-light (R) perception, they are converted to the biologically active Pfr conformer (Rockwell et al. 2006). Pfr is thermodynamically unstable, and it spontaneously converts back to Pr. This process is called thermal reversion and has key importance in the attenuation of light signaling in the dark or under low light conditions (Klose et al. 2020). Furthermore, higher ambient temperature decreases Pfr levels by accelerating thermal reversion, allowing phytochrome B to act as a thermosensor (Jung et al. 2016, Legris et al. 2016, Kerbler and Wigge 2023).

All phytochromes are translocated to the nucleus upon formation of their Pfr conformer and localize to subnuclear complexes (photobodies) where they interact with different proteins, and these steps are necessary for most phytochrome functions (Fankhauser and Chen 2008, Klose et al. 2015, Kim et al. 2023). For example, Pfr phytochromes physically interact with phytochrome-interacting factor (PIF) transcription factors, which have a central role in maintaining skotomorphogenic development, inducing their rapid degradation thus allowing the initiation of photomorphogenesis (Leivar et al. 2008, Shin et al. 2009, Ni et al. 2014). Similarly, photoactivated phyB directly interacts with the BRI1-EMS-SUPPRESSOR 1 (BES1) transcription factor and represses its activity. This is how BES1-promoted brassinosteroid-dependent hypocotyl elongation, which occurs in the dark, is blocked at the onset of light (Sun et al. 2010; Wang et al. 2006, Wu et al. 2019).

Although phytochromes do not possess DNA-binding domains, distinct regions of the genome were identified as binding sites of phyB-containing protein complexes, suggesting that this is a direct step by which light modifies gene transcription (Chen et al. 2014, Jung et al. 2016).

Phytochromes are categorized into two groups based on their stability in light. phyA, the only type I phytochrome, is unstable in R, acts exclusively as homodimers and mediates responses at very low light intensities (very low-fluence responses) and in continuous FR (high-irradiance responses). Type II phytochromes (phyB-phyE) are more stable under irradiation than phyA. They regulate R/FR reversible low-fluence responses, and among them, phyB has the most important role (Nagy and Schäfer 2002). It was demonstrated that phyB, the most abundant and important type II phytochrome, accumulates to higher levels in dark-grown plants than in light-grown plants because the Pfr form of phyB degrades faster than Pr (Ni et al. 2014).

Being the dominant phytochromes for most light responses, phyA and phyB signaling is in the spotlight of phytochrome research. However, studies examining single- and higher-order phytochrome mutants revealed that despite having subtle roles, phyC–phyE do mediate light responses (Strasser et al. 2010, Hu et al. 2013, Sánchez-Lamas et al. 2016). These phytochromes take part in seedling development, flowering induction, shade avoidance and germination responses, but their effects are obscured in the presence of phyA or phyB (Aukerman et al. 1997, Devlin et al. 1998, 1999, Franklin et al. 2003, Halliday and Whitelam 2003, Monte et al. 2003). It was also reported that whereas endogenous phyB and phyD form homodimers, phyC and phyE form obligate heterodimers with phyB and phyD (Sharrock and Clack 2004, Clack et al. 2009, Liu and Sharrock 2013), although other studies showed that phyC and phyE homodimers are functional, indicating that the issue of homo/heterodimerization of these phytochromes needs further investigations (Clack et al. 2009, Ádám et al. 2013, Viczián et al. 2020).

Together with the studies performed using null mutants, overexpression of phyC–phyE was also used to study their functions. Overexpressed and tagged phyC, phyD and phyE are functional photoreceptors and are able to mediate more pronounced light responses than their genomic counterparts (Kircher et al. 2002, Sharrock et al. 2003a, Fernández et al. 2005, Ádám et al. 2013). These responses are also induced by overexpressed N-terminal fragments of these phytochromes, demonstrating their activity in red-induced photomorphogenesis and indicating that their C-terminal domain is not required for signaling (Ádám et al. 2013).

Expression of phyC–phyE fused to fluorescent proteins as chimeras allowed to monitor their intracellular localization. Similarly to phyA and phyB, light irradiation triggers their nuclear accumulation, but only phyC and phyE form photobodies under R irradiation (Kircher et al. 2002, Ádám et al. 2013). A recent study demonstrated that the hyperactive mutant version of phyD can also localize to photobodies during photomorphogenic development, indicating that these structures are linked with active phyD signaling (Viczián et al. 2020).

PhyD appeared late during phytochrome evolution by a Brassicaceae-specific gene duplication of phyB (Mathews 2005, Mathews and McBreen 2008). Although the phytochrome B and D proteins share high-sequence homology, the two phytochromes function differently because phyD accumulates to lower levels and the differences in their protein sequences have signaling consequences. Despite its low expression level, phyD plays a role in the regulation of many different developmental programs, including germination, seedling photomorphogenesis or flowering (Sharrock et al. 2003a, 2003b, Ádám et al. 2013).

To further study the role of phyD and its interaction with other phytochromes, we overexpressed phyD-YFP in different higher-order phytochrome mutant backgrounds. We found that whereas this chimeric protein alone cannot effectively mediate photomorphogenesis, it can cooperate with phyC, and under low temperatures, phyD-YFP accumulates to high levels, aggregates to photobodies and promotes de-etiolation efficiently. Moreover, we found that light signaling mediated by phyB and phyD is similar, and after a short red-light treatment, these photoreceptors are associated with strongly overlapping genetic loci and change transcription of similar target genes. However, our results also suggest that phyD-YFP is a less efficient photoreceptor than phyB because (i) only part of the phyD-YFP pool is photoactivable (ii) and its signaling is less efficient likely because the thermal reversion of phyD-YFP is faster. Conclusively, our results suggest that under specific conditions, phyD can substitute or complement the activity of phyB, thereby increasing the flexibility of plants’ responses to environmental changes.

## Results

### phyC and low temperature are required for efficient induction of seedling photomorphogenesis by phyD-YFP

**Fig. 1:**
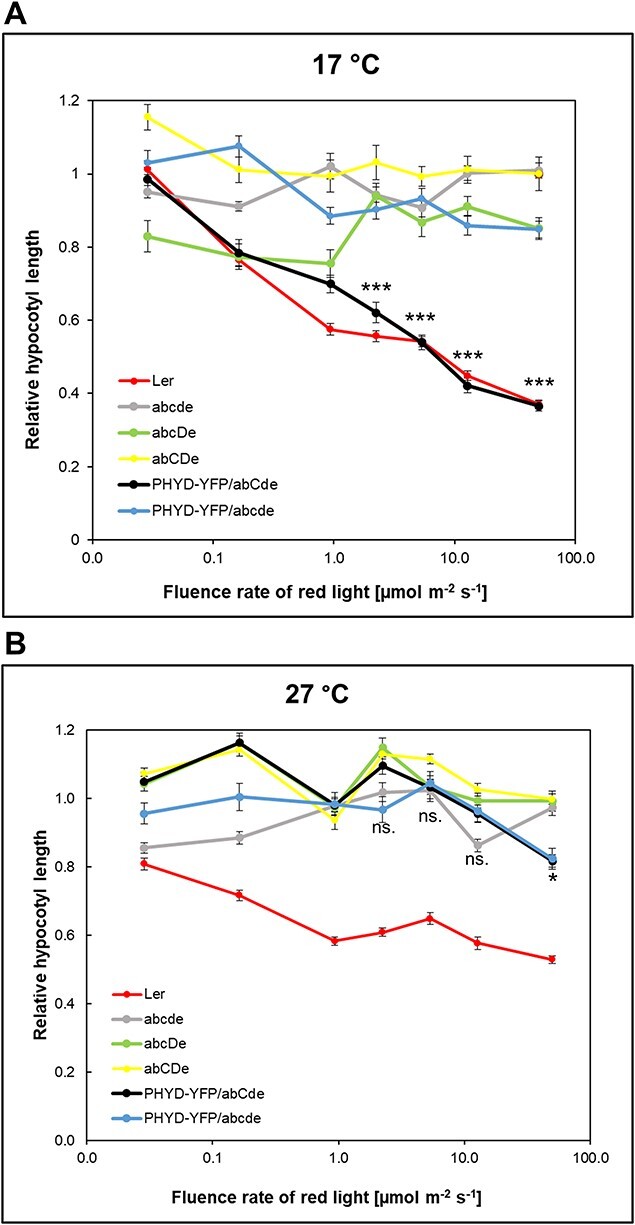
PhyD-YFP modulates photomorphogenesis in the presence of phyC at lower temperatures. Seedlings were grown at either 17°C (A) or at 27°C (B) for 4 d under different fluences of constant R light irradiation. Hypocotyl values relative to the corresponding dark controls are shown. *n* ≥ 30, error bars indicate standard errors. Asterisks denote significant differences between the PHYD-YFP/abCde and the abcde lines at the marked data points (Mann–Whitney *U* test, *P < 0.05; ***P < 0.001; ns.: not significant).

To examine the role of phyD, we expressed phyD-YFP under the control of the strong constitutive *35S* promoter in quintuple phytochrome mutant *A. thaliana* Landsberg *erecta* (*Ler*) ecotype (abcde). The line that expressed phyD-YFP to the highest level (PHYD-YFP/abcde) was selected and used for further studies and crossings. To assess the function of the transgene, we examined its efficiency in the inhibition of hypocotyl elongation response under R irradiation at low and high ambient temperatures (17°C and 27°C, respectively) (**Fig. 1**). The PHYD-YFP/abcde seedlings showed no significant hypocotyl length shortening throughout the red-light fluence rate range, indicating that phyD-YFP alone cannot mediate this response. We crossed the *35S:PHYD-YFP* transgene to a background having no functional phytochromes except phyC, obtaining the PHYD-YFP/abCde transgenic line. Interestingly, PHYD-YFP/abCde inhibited hypocotyl elongation as efficiently as the wild-type (WT) *Ler* line at 17°C, whereas it showed no response at 27°C. Endogenous phyD alone (abcDe) or together with phyC (abCDe) showed no effective photomorphogenic response at either temperature, suggesting that a high phyD-YFP level was required for efficient response (**Fig. 1**). Thus, we concluded that in young seedlings, phyD-YFP can efficiently mediate photomorphogenesis only at low temperature and in the presence of phyC.

### PHYD-YFP protein accumulation depends on the temperature and the presence of phyC

**Fig. 2:**
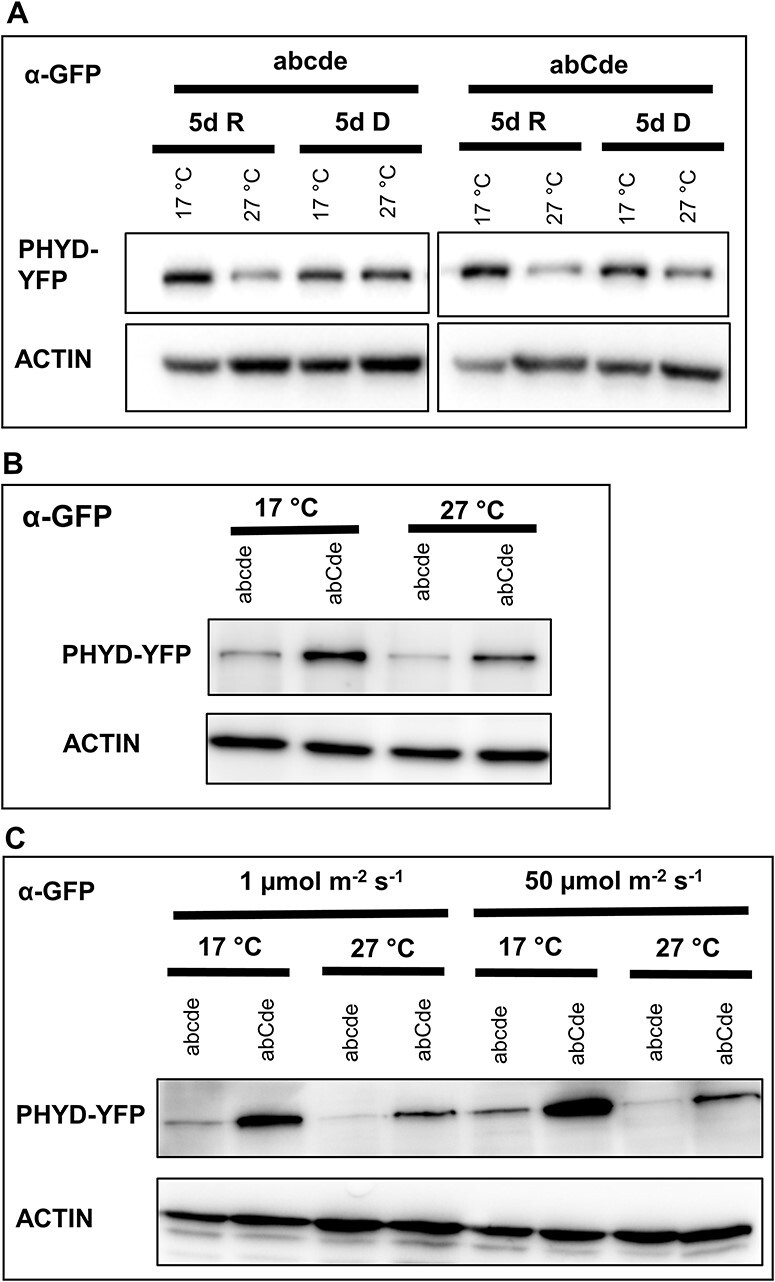
Accumulation of PHYD-YFP depends on the temperature and the presence of phyC in the dark and under constant R irradiation. (A) Seedlings expressing *35S:PHYD-YFP* either in the abcde or in the abCde genetic background were grown in the dark (D) or under constant R irradiation (50 µmol m^−2^ s^−1^) (R) for 5 d at 17°C or 27°C. (B) The same seedlings as in (A) were grown in the dark for 5 d at the indicated temperatures. (C) The same seedlings as in (A) were grown under constant weak or strong R irradiation for 4 d at 17°C or 27°C. Amounts of PHYD-YFP protein are determined by immunoblotting using anti-GFP antibody and ACTIN is used as loading control in each panel.

As photoreceptors are positive regulators of light signaling, their abundance has a direct effect on the intensity of light responses. Therefore, we measured whether the amounts of PHYD-YFP protein correlate with the observed phyD-YFP-dependent phenotypes. We found that in the abcde background, PHYD-YFP accumulates to higher amounts at 17°C than at 27°C in the light- but not in dark-grown plants (**Fig. 2A**), whereas in the abCde background, PHYD-YFP levels were higher at 17°C than at 27°C in both light- and dark-grown plants (**Fig. 2B, C**). Interestingly, the amount of PHYD-YFP was higher under all tested light and temperature conditions in the abCde than in the abcde background (**Fig. 2B, C**). Thus, we concluded that the amounts of phyD-YFP are correlated with photomorphogenic responses, and the highest amount of phyD-YFP and the most pronounced photomorphogenesis were observed in the abCde background at 17°C. Additionally, we found that short red irradiation does not significantly alter the amount of PHYD-YFP (**Supplementary Fig. S1**), but a temperature shift of 3 d results in similar amounts of protein as the growth of 5 d at the same temperature (**Supplementary Fig. S2**).

As we described earlier, in the *Ler* ecotype, in the quintuple phytochrome mutant PHYD-YFP accumulated to higher levels and functioned more efficiently at low temperatures. To exclude the possibility that it is the specific feature of this transgenic line, we tested three independent transgenic plants that express phyD-YFP in the abCdE background (Viczián et al. 2020). We found that PHYD-YFP accumulates light- and temperature-dependently in this background in a similar fashion as in the abCde (**Fig. 2**). We note that the phospho-state of serine 82 of phyD-YFP (S82A: non-phosphorylated, S82S: phospho-mimic) does not affect this accumulation pattern (**Supplementary Fig. S3**). We transformed *35S:PHYD-GFP* into a different *Arabidopsis* ecotype, Wassilewskija (Ws), that lacks functional phyD (PHYD-GFP/Ws) (Aukerman et al. 1997), and then we studied PHYD-GFP expression at 17°C and 27°C. We found that the PHYD-GFP protein accumulated to higher levels at low temperatures in both dark- and R-grown PHYD-GFP/Ws seedlings (**Supplementary Fig. S4**). PHYD-GFP also functioned in a temperature-dependent manner in Ws, as the photomorphogenesis response of PHYD-GFP/Ws was stronger at 17°C but not at 27°C than in the control Ws plants (**Supplementary Fig. S4**).

Taken together, overexpressed phyD accumulates to higher levels and functions more efficiently at low temperatures in both *Ler* and Ws ecotypes when phyC is present.

### Low temperature and phyC are required for photobody formation but not for nuclear localization of PHYD-YFP

**Fig. 3:**
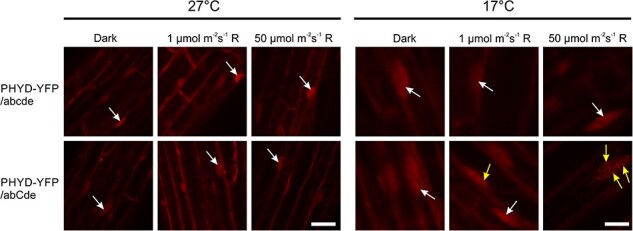
Intracellular localization of phyD-YFP in different genetic backgrounds. *35S:PHYD-YFP* was expressed either in the abcde or in the abCde genetic background. These seedlings were grown at either 17°C or 27°C in the dark or under different fluences of constant monochromatic red-light (R) illumination for 4 d, and then laser scanning confocal microscopy was performed to visualize the YFP-specific signal. White arrows point at selected nuclei containing phyD-YFP in diffuse distribution, whereas yellow arrows mark nuclei having phyD-YFP-containing subnuclear protein complexes (PBs). The signal intensity was set for optimal visualization and not for quantitation. White bars represent 25 µm.

Next, we examined the localization of phyD-YFP under various light and temperature conditions in different backgrounds. We observed nuclear phyD-YFP accumulation in both the abcde and abCde backgrounds at 17°C and 27°C, which is in good correlation with the previously published results obtained at 22°C (Ádám et al. 2013). Photobody formation is correlated to efficient light signaling. Interestingly, we could detect phyD-YFP photobodies in the nucleus only when phyC was present (abCde background) and the seedlings were grown under R at 17°C. Higher light fluence induced the formation of more photobodies than lower light intensity (**Fig. 3**).

Conclusively, both the amounts of phyD-YFP and photobody accumulation are elevated under those conditions when phyD-YFP-dependent signaling is active, i.e. at low temperatures and in the presence of phyC.

### phyD mediates adult development and delays flowering together with phyC at low temperature

**Fig. 4:**
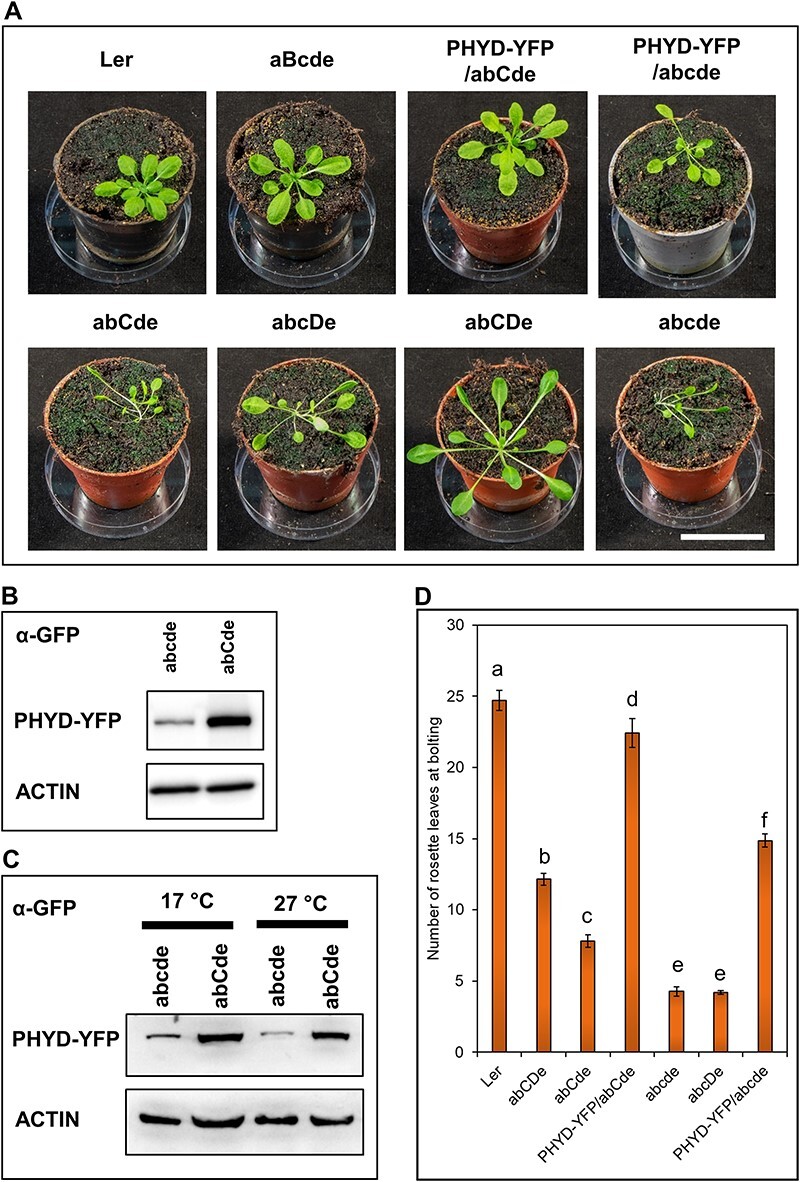
phyD and phyC act cooperatively controlling adult development and delaying flowering at lower temperatures. (A) Plants were grown on soil under a short-day light regime (8 h white light/16 h dark) at 17°C for 60 d. Scale bar indicates 50 mm. (B) The amount of PHYD-YFP protein in the leaves of 60-day-old plants was determined by immunoblotting using anti-GFP antibody. ACTIN was used as a loading control. (C) Plants were grown as in (A) at 22°C for 28 d and transferred at 17°C or 27°C without changing the light regime for 3 d and western blots were performed as described in (B). (D) Plants were grown as in (A) at 17°C. The number of rosette leaves was counted at the time of bolting. Each bar represents the count data of *n* ≥ 40, with means and Poisson confidence intervals (ci: 0.95). Based on non-overlapping confidence intervals, statistically significant differences between the lines are marked with letters.

To investigate how phyD can regulate adult development and flowering, we grew different mutants and PHYD-YFP-overexpressor transgenic plants under a short-day white light regime at 17°C. The quintuple mutant (abcde) shows strong developmental defects under these conditions (**Fig. 4A**), whereas phyD-YFP together with phyC can complement this phenotype to a similar extent as phyB alone (aBcde). In contrast, phyD-YFP alone has a weaker, partial effect. In adult plants, like in seedlings, the amount of PHYD-YFP protein was much higher in the abCde background than in the abcde background (**Fig. 4B**). Furthermore, the shift of 3 d to low temperature results in higher PHYD-YFP protein levels, while the shift to high temperature leads to lower PHYD-YFP protein levels (**Fig. 4C**). These results indicate that similar molecular mechanisms control PHYD-YFP accumulation in seedlings and in adult plants (**Supplementary Fig. S2**). By comparing the adult phenotypes, the effect of endogenous phyD could also be studied. Interestingly, endogenous phyD and phyC together (abCDe) complement the mutant phenotype better than the endogenous phyD (abcDe) or phyC (abCde) alone. Thus, phyC also cooperates with the endogenous phyD in adult plants despite the fact that we could not detect this functional interaction in the seedling state (**Fig. 1**). phyB-phyE delay flowering in *Arabidopsis*, and thus abcde plants flower at a very early stage (Sánchez-Lamas et al. 2016). Flowering time analysis of our mutants confirmed that phyD and phyC synergistically delay flowering at 17°C. We observed partial mutant complementation in PHYD-YFP/abcde plants but full complementation in the PHYD-YFP/abCde line. Similarly to the rosette development phenotype, endogenous phyC and phyD together show better mutant complementation in flowering time tests than these phytochromes alone (**Fig. 4D**). These data corroborate the results of Sánchez-Lamas et al. (2016) demonstrating that phyC and phyD synergistically interact to delay flowering in different *Arabidopsis* ecotypes.

Thus, we conclude that phyD and phyC cooperate in mediating photomorphogenesis in adult plants at low temperatures and, unlike in young seedlings, where overexpression of PHYD is required, this coaction can also be observed in plants that express only endogenous phytochromes.

### PHYD-YFP/abCde mediates similar transcriptional reprogramming pathways as phyB

Phytochrome B alone (aBcde) is able to mediate photomorphogenesis in constant R at both low and high temperatures, whereas PHYD-YFP shows effective response only at 17°C and in the presence of phyC (**Supplementary Fig. S5**). To unravel whether phyB and PHYD-YFP/abCde activate similar or different transcriptional responses, we conducted comparative RNA- and ChIP-seq assays.

**Fig. 5:**
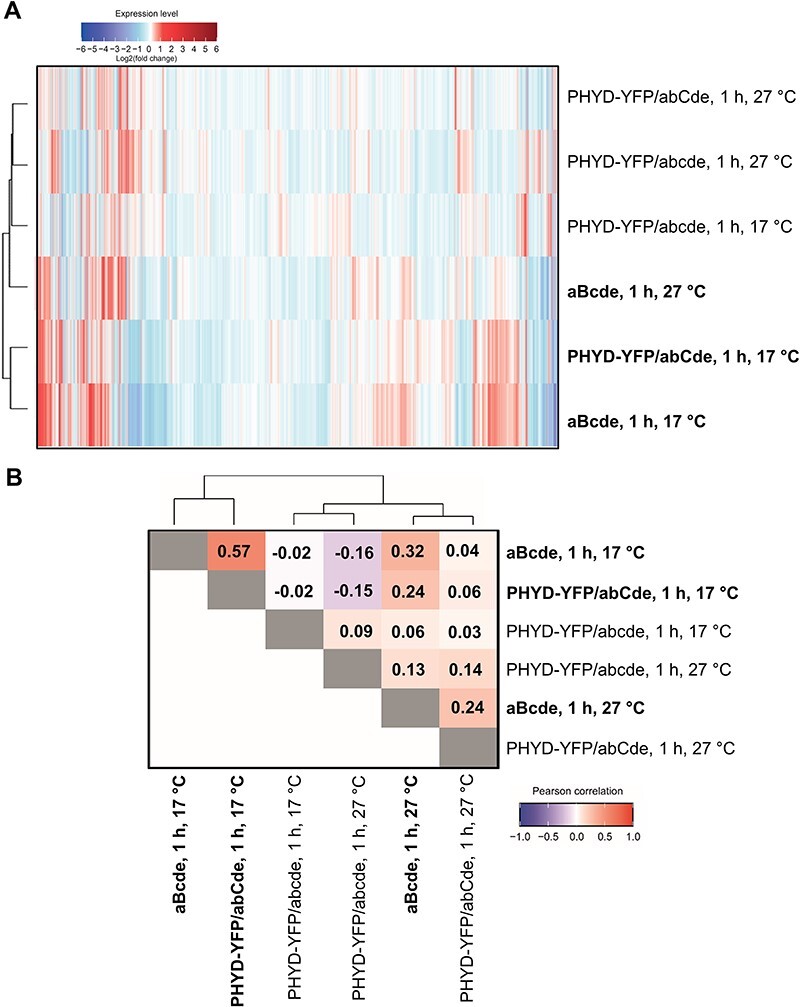
Red-light-induced early transcriptional changes in different *Arabidopsis* phytochrome mutants. (A) Heat map of transcriptome changes in response to 1 h red-light treatment. Hierarchical clustering was calculated based on Euclidean distance with ‘complete linkage’ method. Plants that showed hypocotyl shortening at the given conditions are printed in bold. (B) Pearson’s correlation of transcriptome changes after 1 h red-light treatment. Note that PHYD-YFP/abCde and the corresponding aBcde samples are grouped and their transcriptome changes correlate only at low temperatures.

We performed RNA-seq on samples isolated from dark-grown and R-treated (1 or 24 h) PHYD-YFP/abcde, PHYD-YFP/abCde and aBcde plants grown at either 17°C or 27°C (**Supplementary Fig. S6A**). Principal component analysis indicated that (i) the repeats are well clustered; (ii) the two temperature conditions separate the samples clearly into two major groups and (iii) those lines that show hypocotyl photomorphogenesis and undergo 24 h irradiation are separated well from the others (**Supplementary Fig. S6B**). Heat-map and correlation analyses unraveled that the early (1 h light) transcriptional responses of PHYD-YFP/abCde and aBcde plants are similar at low but not at high temperatures. These samples were grouped on a heat map, and changes in their expression were correlated (Pearson’s *r* = 0.57) only at 17°C (**Fig. 5**). Although the transcriptional changes of PHYD-YFP/abCde and aBcde plants resembled each other at both temperatures after 24 h light, the similarity was higher at low temperature. At 17°C, these samples were grouped on a heat map, and their transcriptional changes showed a strong correlation (Pearson’s *r* = 0.72) (**Supplementary Fig. S7**). Moreover, after 24 h, the overlap between the differentially expressed genes of PHYD-YFP/abCde and aBcde plants was stronger at low temperatures (**Supplementary Fig. S8, Supplementary Table S1**, **Supplementary Datasets S1, S2**). Thus, under these conditions, R induces similar early and late (1 and 24 h) transcriptional responses in aBcde and PHYD-YFP/abCde plants.

**Fig. 6:**
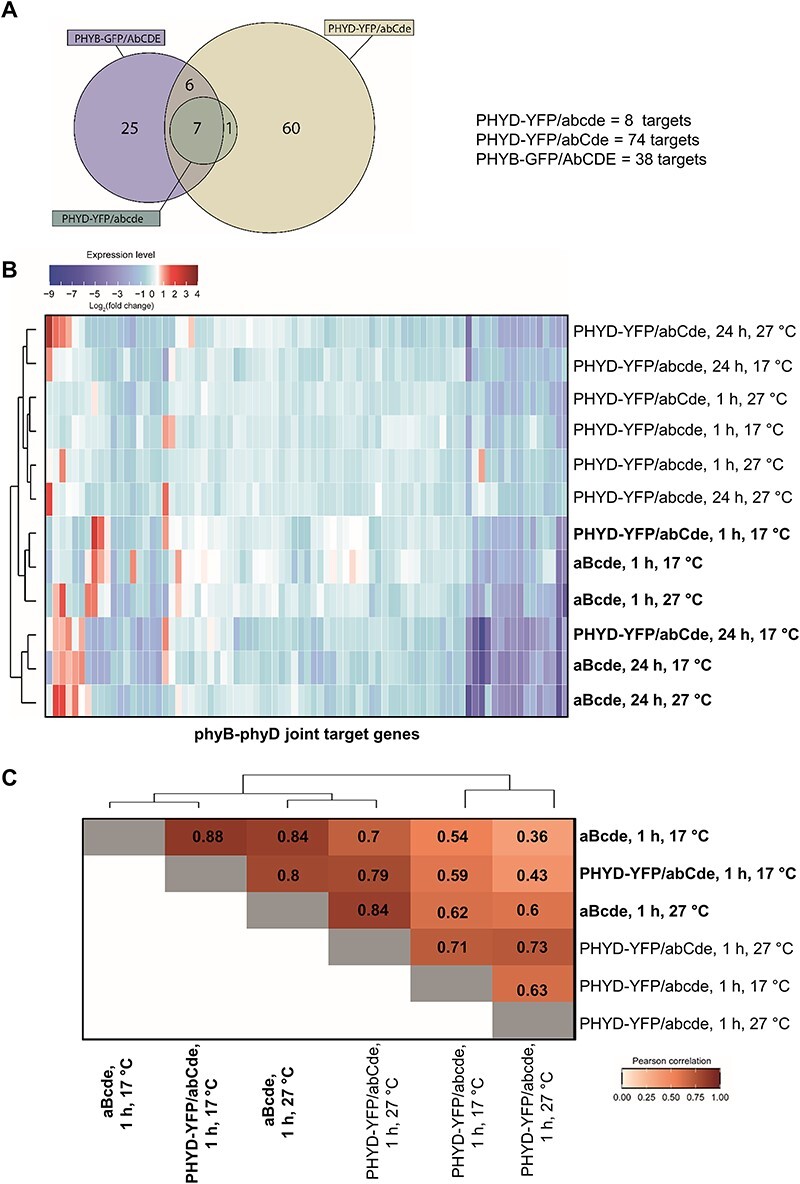
Red-light-induced transcriptional changes of the phyB-phyD joint target genes in different *Arabidopsis* phytochrome mutants. Direct targets of phyB-GFP and phyD-YFP phytochromes were identified in ChIP-seq assays. (A) Venn diagram of direct targets of phyD-YFP (in the abcde and abCde backgrounds) and phyB-GFP (in the AbCDE background) phytochromes. Significant overlap between phyD-YFP and phyB-GFP targets was detected by Fisher’s exact test (two-sided) on a 2 × 2 matrix (*P* = 2.20E-16). (B) Heat map of changes in the expression of the phyB–phyD joint target genes in response to 1 and 24 h red-light treatments. Hierarchical clustering was calculated based on the Euclidean distance with the ‘complete linkage’ method. Plants that showed the photomorphogenic response of hypocotyl shortening under the given conditions are printed in bold. Note that PHYD-YFP/abCde and aBcde samples are grouped only at low temperatures at both time points. (C) Pearson’s correlation of changes in the expression of the phyB–phyD joint target genes in response to 1 h red-light treatment.

To compare the genomic direct targets of phyB and phyD, we conducted ChIP-seq experiments using anti-GFP antibody on samples isolated from PHYD-YFP/abCde, PHYD-YFP/abcde and from a *35S:PHYB-GFP*-overexpressing plant (PHYB-YFP/AbCDE), all of which were grown at 17°C in dark and R-irradiated for 5 min (**Supplementary Fig. S6A**). We selected this short R irradiation because we detected relevant transcriptional changes already after 1 h R and because no ChIP-seq results of type II phytochrome at the very early time point of photomorphogenesis have been reported. Eight, 74 and 38 bound genes were identified in the PHYD-YFP/abcde, PHYD-YFP/abCde and PHYB-GFP/AbCDE plants, respectively (**Fig. 6A**, **Supplementary Datasets S3, S4**, **Supplementary Table S2**). ChIP-qPCR assays performed on dark and R-irradiated samples demonstrated that the binding of phyD-YFP and phyB-GFP to the common target genes (see later) is light-dependent (**Supplementary Fig. S9**). Relevantly, all eight PHYD-YFP/abcde targets were also present in the PHYD-YFP/abCde. Moreover, they were the most over-represented targets in the PHYD-YFP/abCde sample (**Supplementary Dataset S4**). These results indicate that phyD-YFP alone can only bind the strongest targets (showing the highest fold enrichment in the PHYD-YFP/abCde), whereas it binds several other genes in the presence of phyC. Seven out of the eight PHYD-YFP/abcde targets were also found in the PHYB-GFP/AbCDE ChIP-seq sample (targets bound in all three samples are referred to as common targets). In addition to the seven common targets, only six additional genes were over-represented in both the PHYD-YFP/abCde and PHYB-GFP/AbCDE ChIP samples (**Fig. 6A**), suggesting that phyD-YFP and phyB-GFP bind different (although significantly overlapping) gene sets. As PHYD-YFP/abCde and phyB induced transcriptional changes correlated at 17°C after 1 h light, this unexpected finding apparently suggests that the two phytochromes activate similar transcriptional responses by binding relatively different gene sets. Alternatively, they bind more similar gene sets but with different efficiencies and/or dynamics; therefore, CHIP-seq at a given time point (5 min red light) identifies only a fraction of genuine common targets. To distinguish between these two possibilities, we combined the genes bound by PHYD-YFP/abCde and PHYB-GFP/AbCDE in a single list (named phyB-phyD joint targets) and then analyzed their expression in our RNA-seq (**Supplementary Dataset S5**). We assumed that phytochrome binding leads to relevant changes in target gene expression. If phyD-YFP (in the abCde background) and phyB bind very similar gene sets, the expression of phyB–phyD joint targets will be similar in the aBcde and PHYD-YFP/abCde samples. Indeed, at low temperatures the expression of phyB-phyD joint targets altered very similarly in these plants already after 1 h irradiation (**Fig. 6B, C**), and after 24 h the transcriptional changes of the phyB-phyD joint targets were almost identical (Pearson’s *r* = 0.93) in the PHYD-YFP/abCde and aBcde plants (**Supplementary Fig. S10A**). These results suggest that phyB-GFP and phyD-YFP bind strongly overlapping gene sets and modify the expression of these genes very similarly (**Fig. 6A**).

The heat map also shows (**Fig. 6B**) that most of the phyB–phyD joint targets were already downregulated after 1 h R treatment in both the aBcde and PHYD-YFP/abCde samples and they were still repressed after 24 h, indicating that early DNA binding by phyB and phyD predominantly leads to quick and stable repression of the target genes. Next, we studied the expression changes of the phyB-phyD joint targets in the RNA-seq of aBcde plants grown at 17°C. Out of the 99 phyB-phyD joint targets, 32 showed significantly altered, mainly reduced expression. Thirteen targets were downregulated at both time points and 14 only after 24 h R irradiation, whereas only three were up-regulated and two mixed regulated (**Supplementary Fig. S10B**, **Supplementary Dataset S6**). Red-light response and transcription GO categories were over-represented among the phyB–phyD joint targets, which were significantly downregulated (**Supplementary Dataset S6**). These results that red light leads to quick phytochrome binding to light-responsive and transcription-related genes, which results in rapid and stable changes in their expression, indicate that binding of phytochromes to genomic locations at the onset of light plays a key role in photomorphogenesis-related transcriptional reprogramming.

Collectively, under low temperatures phyD-YFP in abCde activates similar signaling as phyB, and they bind to a similar gene set and trigger strongly correlating changes in gene expression.

### Comparisons of phyB and phyD-YFP signaling

**Fig. 7:**
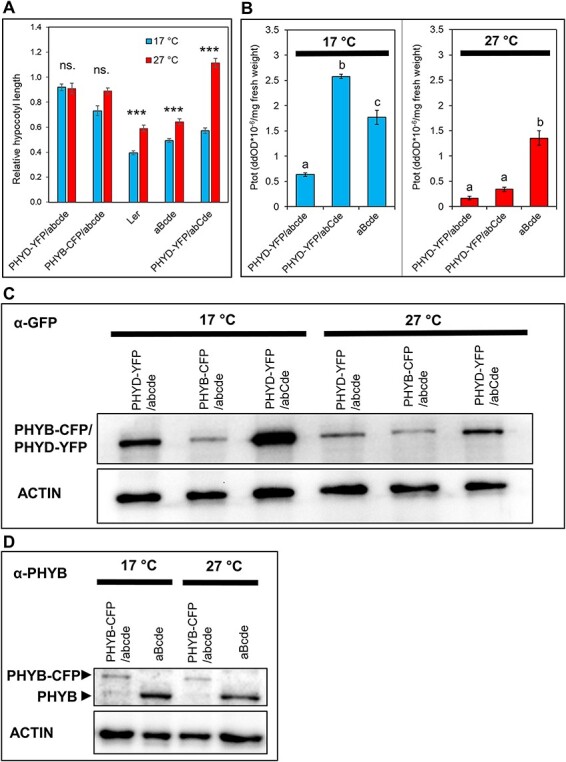
Accumulation and function of phyD-YFP depends on the temperature and the presence of phyC. Seedlings were grown under constant R irradiation (50 µmol m^−2^ s^−1^) for 4 d at 17°C or 27°C on norflurazon-containing medium. (A) Hypocotyl length values are normalized to the corresponding dark control. *n* ≥ 30, error bars depict standard errors. Asterisks denote significant differences between the 17°C and 27°C treatment (Mann–Whitney *U* test,***P < 0.001, ns.: not significant). (B) Total photoconvertible phytochrome amounts (Ptot) are normalized to fresh weight. *n* ≥ 5 error bars indicate standard errors. We found no equal variance across 17°C samples (Levene’s test, *P* < 0.001); hence, we performed Welch’s ANOVA (*P* < 0.001) and Games-Howell post hoc test to see significant differences at the 0.05 level. Significant differences between the 27°C samples were analyzed at the 0.05 level and were calculated with ANOVA (*P* < 0.001) followed by Tukey’s *post hoc* test. (C) The amounts of PHYD-YFP and PHYB-CFP proteins are determined by immunoblotting using anti-GFP antibody. ACTIN is used as a loading control. (D) PHYB-CFP and endogenous PHYB are detected by anti-PHYB antibodies. ACTIN is used as a loading control.

The efficiency of signaling in R correlates better with the amount of active Pfr than with total phytochrome levels. Although phyD-YFP accumulated to high levels in the abCde seedlings, we were unable to determine the relative phyD-YFP Pfr levels by *in vivo* spectroscopy due to the overall low detectable signal for the total amount of photoreversible phytochrome (Ptot). In order to understand the characteristics of phyD signaling, we analyzed total protein and Ptot amounts of phyD-YFP and phyB to assess their biologically active and non-active protein pools. As signaling of phyD-YFP in abCde was comparable to that of phyB in aBcde at all fluence rates at 17°C (**Fig. 1**), we assumed that phyD-YFP (at least in the presence of phyC and at low temperature) and phyB are similarly efficient photoreceptors. Indeed, Zn-blot assays, which detect chromophore-bound proteins, indicated that at low temperatures, phyD-YFP and phyB bind chromophore with similar efficiency and that phyC is not requested for efficient chromophore binding of phyD-YFP (**Supplementary Fig. S11**). Next, we wanted to compare the total protein (western-blot) and Ptot levels (*in vivo* spectroscopy) in PHYD-YFP/abcde, PHYD-YFP/abCde and aBcde plants. For Ptot measurements, R-illuminated seedlings were grown on a bleaching herbicide (norflurazone) containing media to avoid chlorophyll accumulation, which can interfere with *in vivo* spectroscopic measurements of phytochromes (Klose et al. 2020). We tested the effect of norflurazone on signaling and protein accumulations and found that the inhibition of hypocotyl elongation in R showed a similar pattern in the presence of the herbicide as in the original phenotyping assay (**Figs. 1**, **7A**); phyD-YFP mediated efficient signaling only at 17°C in the abCde background, whereas phyB (aBcde) showed an effective response at both temperatures (although giving better photomorphogenic response at 17°C). Moreover, PHYD-YFP protein accumulated to the highest levels in the abCde background and at low temperatures regardless of the presence or absence of norflurazone (**Figs. 2**, **7C**). Thus, norflurazone treatment does not interfere with either the light response or the PHYD-YFP protein levels. We found that the total protein and Ptot levels of phyD-YFP correlated well and that at 17°C the Ptot level of phyD-YFP was much higher in the abCde background than in the abcde background (**Fig. 7B**) resembling the difference in the amount of total PHYD-YFP proteins (**Fig. 7C**). Moreover, the total protein and the Ptot levels of phyD-YFP were strongly reduced at 27°C in both backgrounds (**Fig. 7B, C**).

Next, we compared the Ptot levels of phyD-YFP and phyB and their R responses to assess the signaling efficiency of the photoreversible photoreceptors. At 17°C, the Ptot of phyD-YFP in abCde is significantly higher than phyB Ptot and they activated similar photomorphogenic responses. Thus, under the strong continuous R, at low temperatures and in the presence of phyC, photoreversible phyD-YFP is a less efficient photoreceptor than phyB. The Ptot of phyD-YFP in both backgrounds was strongly reduced at high temperatures (27°C), whereas the Ptot of phyB was nearly unaffected by temperature (**Fig. 7B**). In line, only phyB activated photomorphogenesis at high temperatures (**Figs. 1**, **7A**). We do not know whether photoreversible phyD-YFP in abCde at high temperatures signals less efficiently than phyB or inefficient signaling is only due to the low phyD-YFP Ptot levels.

Finally, we estimated the ratio of photoconvertible and non-photoconvertible proteins by comparing the total protein and the Ptot amounts of phyD-YFP and phyB. As anti-PHYB antibody does not identify PHYD-YFP, a reference transgenic plant that expresses phyB-CFP as the only phytochrome (phyB-CFP/abcde) was used. As phyB-CFP can be detected by both anti-GFP and anti-PHYB antibodies, it allowed us to indirectly compare the amounts of phyD-YFP and phyB protein. We found that at 17°C, the total phyD-YFP protein amount in abCde was much higher than the endogenous phyB in aBcde (**Fig. 7C, D**), and this difference was higher than the difference between their Ptot amounts, while at 17°C, phyD-YFP/abCde and phyB induced similar photomorphogenic response. At 27°C, the phyD-YFP protein amount in abCde was comparable to the phyB in aBcde, but its Ptot level was much lower and could not mediate signaling (**Fig. 7**). These data suggest that a significant amount of PHYD-YFP is present in non-photoreversible, likely biologically inactive forms at both temperatures.

Taken together, our results indicate that at low temperature (i) phyD-YFP accumulates to high levels in R; (ii) phyD-YFP binds chromophore; (iii) phyD-YFP presents in inactive (non-photoconvertible) and biologically active (photoconvertible) pools and (iv) in the presence of phyC the photoconverted phyD-YFP initiates signaling in continuous R with less efficiency than phyB.

**Fig. 8:**
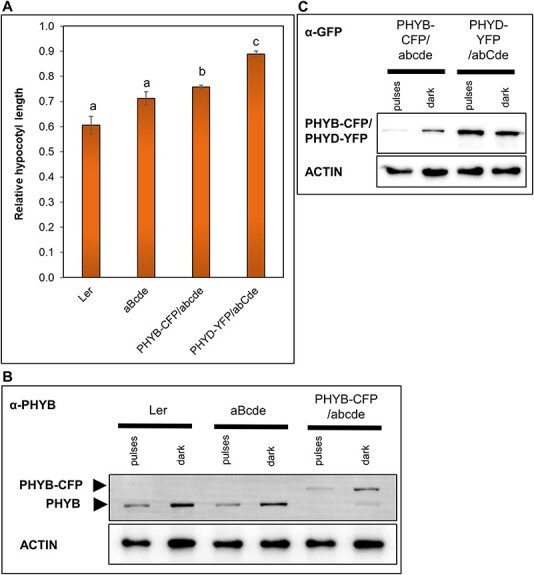
PhyD-YFP accumulates to higher levels but initiates lower intensity signaling than phyB-CFP under R light pulses. Seedlings were grown at 17°C for 4 d in darkness or in darkness that was interrupted by 1-min-long R pulses (50 µmol m^−2^ s^−1^) in every 3 h. (A) Hypocotyl length values are normalized to the corresponding dark controls. *n* ≥ 30, error bars depict standard errors. Significant differences between the samples are marked with letters and were calculated at the 0.05 level using ANOVA (*P* < 0.001) followed by Tukey’s post hoc test. (B) PHYB-CFP and endogenous PHYB are detected by anti-PHYB antibodies. ACTIN is used as a loading control. (C) The amounts of PHYD-YFP and PHYB-CFP proteins are determined by immunoblotting using anti-GFP antibody. ACTIN is used as a loading control.

We carried out all seedling photomorphogenesis experiments in continuous red light where the signaling efficiency is less affected by the thermal reversion rate. To compare the thermal reversion rates of phyB and phyD-YFP photoreceptors, we conducted light pulse experiments. PHYD-YFP/abcde, PHYD-YFP/abCde, aBcde and PHYB-CFP/abcde plants were grown at 17°C in darkness, but in every 3 h, we added 1 min-long saturating red-light pulses. Under these conditions, signaling depends not only on the amount of photoreversible photoreceptors but also on Pfr stability. In the dark, thermal reversion reduces the biologically active Pfr form thereby tuning the level of active signaling between the light pulses. **Fig. 8** demonstrates that under light pulses, phyD-YFP cannot mediate effective light signaling despite that it accumulates to very high levels in the presence of phyC. On the contrary, phyB and phyB-CFP activate photomorphogenic response efficiently and the amounts of their protein are dramatically reduced by the light pulses. These data that phyB mediates efficient signaling at both continuous and pulse lights, while phyD-YFP functions efficiently only at continuous light indicate that the Pfr stability of phyD-YFP is significantly weaker (**Figs. 1**, **8**).

Collectively, our data suggest that phyD-YFP is a less efficient photoreceptor than phyB because (i) a significant part of phyD-YFP proteins are present in non-photoconvertible form; (ii) the photoconvertible form is less active than phyB and (iii) the thermal reversion of phyD-YFP is faster.

## Discussion

### phyD activity is stimulated by phyC and low temperature

We examined the role of phyD-YFP in transgenic seedlings in which phyD-YFP could accumulate to high levels (**Figs. 2**, **7**, **Supplementary Figs. S3, S4**). Our data corroborate and extend previous results (Sharrock and Clack 2004, Sánchez-Lamas et al. 2016) by showing that phyD-YFP alone is a functional but inefficient photoreceptor, which in ChIP-seq assay binds only the strongest phytochrome D target genes. In adult plants, it only partially complements the quintuple phytochrome mutant phenotype and delays flowering moderately (**Figs. 4**, **6**). Relevantly, we also show that, independently of the developmental stage, phyD-YFP operates much more effectively at low temperatures and when functional phyC is present. In this case, phyD-YFP formed photobodies and efficiently mediated photomorphogenesis at 17°C, whereas at 27°C, it failed to produce photobodies and complement the seedling phenotype (**Figs. 1**, **3**). We also detected functional coaction of phyD-YFP and phyC under low temperatures in adult plants where they promote rosette growth and can delay flowering time, thereby complementing these quintuple phytochrome mutant phenotypes quite efficiently. In contrast to young seedlings, in adult plants endogenous phyC and phyD can also mediate detectable cooperative signaling, although less efficiently than overexpressed phyD-YFP with endogenous phyC (**Figs. 1**, **4**). We suspect that in young seedlings a higher amount of phyD is required for photomorphogenic response than in adult plants.

### High-level accumulation of phyD is required for efficient R responses

The expression level of photoreceptors strongly impacts on light responses. Indeed, we found that phyD-YFP expression and the efficiency of light signaling were correlated and that the overexpressed phyD-YFP complemented better the phytochrome mutant phenotypes than the endogenous phyD and high phyD-YFP protein level was associated with efficient signaling. PhyD-YFP expression reaches higher levels (i) at 17°C than at 27°C and (ii) in the presence of phyC than in its absence (**Fig. 2**). Thus, phyD-YFP accumulated to the highest level at 17°C in the presence of phyC.

It is not known how low temperature and phyC stimulate phyD-YFP accumulation. PhyB can be degraded in a Pfr-dependent, activity-coupled manner. However, light signaling of phyD-YFP is weak at high temperatures, and hence it is very unlikely that Pfr-dependent, activity-coupled degradation leads to low phyD-YFP levels unlike in the case of phyB (Ni et al. 2014). Moreover, the results of R pulse experiments suggest that the thermal reversion of phyD-YFP is fast even at low temperatures (also see below), and thus it is unlikely that accelerated reversion plays an important role in weak phyD-YFP signaling at 27°C. We assume that phyD is a thermosensitive protein that is unstable at high ambient temperatures. Alternatively, temperature- and light-dependent binding of phyD to so far unknown phytochrome-interacting proteins may regulate its protein stability.

PhyC elevates phyD-YFP protein levels and stimulates phyD-YFP-mediated light signaling (**Figs. 1**, **2**, **Supplementary Fig. S1**). It was proposed that phyC and phyD form functional heterodimers *in vivo* especially in the absence of phyB (Sharrock and Clack 2004, Clack et al. 2009). If such heterodimers are more stable and/or active than phyD homodimers, this could explain how phyC enhances phyD levels and/or signaling (Sharrock and Clack 2004, Clack et al. 2009). Alternatively, the signaling pathways of these receptors might interact synergistically and thereby intensify light signaling. The effective coaction of phyD-YFP and phyC in the ChIP-seq assay indicates that direct receptor interaction or very early light-induced signaling events are involved in mediating this cooperation. Independent of the mechanism, we note that the positive effect of phyC appears to be specific for phyD-YFP, as phyE-YFP signaling and accumulation are not modified by phyC (**Supplementary Fig. S12**).

### Significant part of PHYD-YFP accumulates in non-photoconvertible form

We found that although both phyB and phyD-YFP bind chromophores at low temperatures, a significant portion of PHYD-YFP proteins are present in non-photoconvertible form. At 17°C, phyD-YFP accumulated to much higher levels than phyB and had slightly higher Ptot values than phyB, but the two photoreceptors activated similar light responses. At 27°C, phyB (in aBcde) and phyD-YFP (in abCde) proteins accumulate to comparable amounts, whereas the Ptot of phyB is much higher and only phyB can mediate signaling (**Fig. 7**). It is possible that YFP-tagged phyD folds less efficiently than the endogenous phyD. Alternatively, the folding of phyD is more error-prone than the closely related phyB. We also speculate that phyD folding is temperature-sensitive and at high temperatures the improperly folded proteins are rapidly degraded explaining the temperature-dependent accumulation and signaling.

### Pfr stability of phyD-YFP could limit signaling at low temperature

Under strong red-light irradiation pulses, phyD-YFP did not activate photomorphogenic response even at low temperatures and in the presence of phyC, whereas phyB mediated efficient light signaling (**Fig. 8**). The most straightforward explanation is that the thermal reversion of phyD Pfr is faster than that of phyB Pfr under these conditions. Alternatively, the strong light pulses that saturate phyB Pfr conversion are not sufficient for efficient Pfr conversion of phyD-YFP. However, our ChIP-seq revealed that a short 5-min R light pulse was sufficient to induce efficient DNA binding of phyD-YFP; thus, we think that faster thermal reversion of phyD-YFP is the likely explanation. Faster thermal reversion can also explain why photoreversible phyD-YFP mediates light signaling less efficiently than phyB.

Interestingly, phyE compared to phyB has extreme Pfr stability (Viczián et al. 2020). It is interesting to speculate that phyD and phyE, both of which have evolved from the ancient phyB, can fulfill markedly different functions based on their different thermal reversion rates. phyD, whose Pfr rate is fast, could contribute to red-light signaling under constant irradiation, whereas phyE, whose Pfr is extremely stable, could play an important role in maintaining phytochrome signaling in the dark or under dim light conditions. Comparative analyses of phyB, phyD and phyE could reveal the different factors that are responsible for the markedly different Pfr accumulation and thermal reversion characteristics of these phytochromes.

### Different thermosensing of phyD and phyB

Plants respond to high ambient temperatures by exhibiting characteristic morphological changes (thermomorphogenesis). PhyB plays a critical role in thermomorphogenesis because its activity is strongly influenced by thermal reversion rate: at higher temperatures, the thermal reversion of phyB is faster, and hence signaling by phyB is less effective (Jung et al. 2016, Legris et al. 2016). Our data show that phyD also operates less efficiently at high temperatures, and thus it can be regarded as a thermosensor because it ‘receives and converts temperature stimuli into recognizable molecular signals’ (Kerbler and Wigge 2023). Notwithstanding, the molecular bases of phyB- and phyD-mediated thermosensing appear to be different. Our light pulse data demonstrate that phyD Pfr accumulation is low, most probably due to enhanced thermal reversion, and limits signaling efficiency already at 17°C. This observation indicates that phyD thermosensing might not be based on the temperature-dependent thermal reversion rate. Instead, we suggest that phyD functions inefficiently at high temperatures because enhanced temperature leads to reduced amounts of phyD protein in seedlings and in adult plants (**Figs. 2**, **4C**, **7**; **Supplementary Fig. S2**). As phyB and phyD activate similar transcriptional changes (see the next section), the reduced phyD activity at high temperatures could have an importance in fine-tuning phytochrome-regulated thermomorphogenic responses.

### PhyD and phyB phytochromes initiate similar transcriptional reprogramming

By combining RNA- and ChIP-seq assays, we demonstrated that when phyD signaling is efficient (at 17°C in the presence of phyC), it activates similar light-induced transcriptional reprogramming as phyB. ChIP-seq results and expression analyses of the direct targets unraveled that phyB and phyD bind to overlapping gene sets and modify their expression similarly, whereas RNA-seq revealed that they trigger similar transcriptome changes at the early (1 h) time point and even more so at the late (24 h) time point (**Figs. 5**, **6**). We also examined the association of phyB and phyD with genomic targets. PhyD, like phyA and phyB, is able to selectively associate with target genes in a light-dependent manner (**Supplementary Fig. S9**), suggesting that this is a universal mode of action for phytochromes to regulate gene expression (Chen et al. 2014, Legris et al. 2016).

As phytochromes do not directly bind DNA, we postulate that activated phytochromes rapidly interact with functioning transcription factors and modify the expression of the transcription factor-bound target genes. We also assume that this early phytochrome-mediated transcriptional regulation that occurs immediately after the onset of light plays a critical role in light-induced transcriptional reprogramming leading to the developmental transition from skotomorphogenesis to photomorphogenesis. Indeed, we found that after only 5 min red light phyD- and phyB-bound target genes and the expression of these target genes were intensively modified (**Fig. 6**, **Supplementary Fig. S10**). Photomorphogenesis-related GO categories (e.g. response to red light and brassinosteroid or transcription regulation) were over-represented among the phyB–phyD joint targets, indicating that the binding was selective (**Supplementary Dataset S5**). Furthermore, most of the targets were rapidly and stably downregulated (**Fig. 6**, **Supplementary Dataset S6**). During photomorphogenesis, light-activated phytochromes bind and inactivate the negative regulators of photomorphogenesis such as the PIF and BES1 transcription factors (Leivar et al. 2008, Ni et al. 2014, Wu et al. 2019). Our data support these existing models, and we speculate that our experimental conditions allowed us to create a molecular ‘snapshot’ of one of the earliest events of photomorphogenic development, when photoactivated phyB and phyD rapidly bind to the DNA-associated functioning PIFs and BES1 and repress the expression of their target genes, thereby initiating transcriptional reprogramming. In line with this assumption, the light-regulated direct PIF and BES1 targets (Yu et al. 2011, González-Grandío et al. 2022) were significantly over-represented among the phyB-phyD joint targets. Interestingly, the direct targets (Burko et al. 2020) of ELONGATED HYPOCOTYL 5, the key positive regulator transcription factor of photomorphogenesis, were under-represented among the phyB–phyD joint targets (**Supplementary Fig. S13**, **Supplementary Dataset S6**). This indicates that our experimental conditions clearly identified early molecular events of photomorphogenesis that are separated from those changes that are mediated by other factors after prolonged irradiation.

We found that phyD alone can bind only a few strong joint target genes, but the presence of phyC considerably expands its genomic target list. Although in our ChIP-seq assay just a portion of the identified target genes were bound directly by both phyB and phyD, the expression of most of the direct targets are regulated similarly by both phytochromes (**Fig. 6**). This discrepancy between the ChIP-seq and transcriptome results might be caused by the use of PHYB-GFP/AbCDE plants for ChIP-seq, and thus overexpressed phyB-GFP acted together with other phytochromes, whereas phyB was the only active phytochrome in aBcde plants that were used for RNA-seq. Alternatively, the different length of light irradiation before sample collection for ChIP-seq and transcriptome analysis due to methodological reasons or by the altered sensitivity of these methods can be responsible for the discrepancy.

### General conclusions

We identified an interesting aspect of the organization of phytochrome signaling. Namely, we showed that under specific circumstances (continuous light, low temperature) overexpressed phyD in the presence of active phyC very efficiently substitutes for phyB. This finding is somewhat surprising, as it is generally accepted that phyB dominates R signaling in light-grown plants. It is tempting to speculate that further manipulation of phyC–phyE signaling pathways could reveal additional novel modes of action by which these photoreceptors increase the flexibility of plants to adapt to rarely occurring environmental conditions.

## Materials and Methods

### Plant materials and growth conditions

Phytochrome mutants in *the A. thaliana* (L) *Ler* ecotype were obtained by crossings and, for simplifying the text, were abbreviated as follows: abcde (*phyA-201phyB-1phyC-1phyD-1phyE-1*), abCde (*phyA-201phyB-1phyD-1phyE-1*), abcDe (*phyA-201phyB-1phyC-1phyE-1*), abCDe (*phyA-201phyB-1phyE-1*) and aBcde (*phyA-201phyC-1phyD-1phyE-1*) (Hu et al. 2013).

For immunoblot and microscopy assays, seeds were surface sterilized, placed on half-strength MS agar plates and kept at 4°C for 4 d. After 6 h of white light irradiation (22°C, 100 μmol m^−2^ s^−1^, Lumilux XT T8 L 36 W/865 fluorescent tubes; Osram, Munich, Germany), they were grown at different red light (660 nm, Snap-Lite LED light source; Quantum Devices, Barneveld, USA) and temperature conditions for 4 d as described at each experiment. For hypocotyl measurements, seeds were sown on four layers of wet filter paper and kept at 4°C for 4 d and germination was induced with 6 h of white light irradiation at 22°C. Seedlings were grown either at 17°C or at 27°C under different intensities of R or in darkness. Measurement of hypocotyl length and data processing were described previously (Ádám et al. 2013, Dobos et al. 2019).

For *in vivo* spectroscopy, seedlings were surface sterilized, placed on half-strength MS agar plates containing 5 µM norflurazon and kept at 4°C for 4 d. After 6 h of white light irradiation, they were grown for 4 d in R (660 nm LED, 50 µmol m^−2^ s^−1^) at 17°C or 27°C.

### Molecular cloning and generation of transgenic lines

*35S:PHYB-GFP* in *phyB-9* (PHYB-GFP/AbCDE) was described (Medzihradszky et al. 2013). Construction of the *35S:PHYD-GFP* and *35S:PHYD-YFP* has also been described (Kircher et al. 2002, Ádám et al. 2013). *PHYB:PHYB-CFP* was generated as follows: *PHYB* promoter as *Hind*III-*Bam*HI, *PHYB* coding sequence as *Bam*HI-*Stu*I and *CFP-HA* coding sequence as *Sma*I and *Sac*I fragments were inserted into the *pROK2* vector.

*Arabidopsis thaliana* (L.) plants were transformed by the Agrobacterium-mediated floral dip method (Clough and Bent 1998). Transgenic seedlings expressing the fusion proteins were selected by their resistance to hygromycin. Independent homozygous lines expressing one copy of the transgene were selected for further analysis.

### Confocal microscopy

Confocal laser scanning microscopy was performed on the upper hypocotyl cells of 4-day-old seedlings using a Leica SP5 AOBS microscope (Leica, Wetzlar, Germany) using the HC PL APO 20× (NA:0.7) objective lens: with 400 Hz sampling speed, 3× line averaging, 200 μm pinhole and excitation at 514 nm for YFP. The spectral emission detectors were set to 545–582 nm.

### Flowering time measurement

Seeds were sown on soil and kept at 4°C in the dark for 1 week. The pots were transferred to a short-day light regime (8 h light/16 h dark) at 17°C. Flowering time was recorded as the number of rosette leaves at the time when inflorescences reached 1 cm height. Flowering time experiments were repeated three times using 30–40 plants per genotype.

### Total protein isolation, immunoblot and zinc blot assays

Total plant protein extract preparation and immunoblot analysis were performed as previously described (Vanhaelewyn et al. 2019). We used anti-GFP (Clontech, Mountain View, California, USA), anti-ACTIN (Sigma-Aldrich, Burlington, Massachusetts, USA), anti-PHYB (Agrisera, Vännäs, Sweden), primary antibodies and Polyclonal Swine Anti-Rabbit Immunoglobulins/HRP (Dako, Glostrup, Denmark) and Goat Anti-Mouse IgG Peroxidase Conjugated antibody (Invitrogen, Waltham, MA, USA) secondary antibodies. The signals were visualized using Immobilon Western HRP Substrate (Millipore, Burlington, MA, USA) according to the manufacturer’s recommendations using an iBright FL1500 (ThermoFischer Scientific, Waltham, MA, USA) instrument.

For Zinc-blot assays, native total protein extracts from 4-day-old dark-grown seedlings were used. Seedlings were ground in liquid nitrogen and proteins were extracted using 2 ml/g tissue extraction buffer [100 mM NaPO_4_ pH 7.8; 400 mM NaCl; 1 mM KCl; 1 mM EDTA; 1% (w/v) PEG4000; 0.5% (v/v) Triton X-100; Protease inhibitors]. Extracts were cleared by centrifugation and samples were heated for 5 min in 5× SDS-PAGE sample buffer [250 mM Tris-HCl pH 6.8; 10% (w/v) SDS; 50% (v/v) glycerol; 0.01% (w/v) bromophenol blue; 10 mM DTT]. Two-hundred-microgram protein per samples were loaded onto an SDS-PAGE gel containing 1 mM zinc acetate. SDS-PAGE running buffer [25 mM Tris; 192 mM glycin; 0.1% (w/v) SDS] also contained 1 mM zinc acetate. Zinc-stained gels were imaged on a UV-transilluminator with a red filter–equipped camera system. Gels were stained with coomassie blue stain afterward and photographed.

### In vivo spectrophotometry

Ptot was measured in seedlings using a dual wavelength ratiospectrophotometer (Klose 2019). Seedlings were harvested in green light and placed into cuvettes and fresh weight was determined before the measurement. Ptot was determined as ddOD from OD measurements at 730 nm (highest Pfr absorbance) and 800 nm (reference wavelength) after actinic R (660 nm laser diode (LD), 1,000 µmol m^−2^ s^−1^) and FR (730 nm LD, 1,000 µmol m^−2^ s^−1^) irradiation, respectively, which is used to induce phytochrome photoconversion. Ptot was normalized to seedling fresh weight.

### Accession numbers

The accession numbers are PHYB, AT2G18790; PHYC, AT5G35840 and PHYD, AT4G16250.

### ChIP-seq analysis and ChIP qPCR assays

Surface-sterilized seeds were sowed on half-strength MS plates and stratified for 3 d at 4°C. Germination was induced by white light treatment for 6 h, then the plates were incubated in darkness for 6 d at 17°C. Following a red-light pulse at a 50 µmol m^−2^ s^−1^ fluence rate for 5 min, seedlings were fixed in 1% (v/v) formaldehyde solution. For generating non-irradiated samples for ChIP-qPCR assays, parallel sets of plants were kept in darkness until fixing in formaldehyde. The ChIP protocol by Werner Aufsatz (https://www.epigenome-noe.net/researchtools/protocol.php_protid=13.html) was applied with the following modifications. Chromatin samples were sonicated on ice six times for 10 s using a Vibra Cell sonicator (Sonics & Materials, Danbury, CT, USA) at 10% power. Sonicated and diluted chromatin samples were pre-cleared by 20 µl (bed volume) of binding control agarose beads (ChromoTek, Planegg, Germany) for 1 h at 4°C. Chromatin was precipitated using 12.5 µl GFP-Trap agarose beads (ChromoTek, Planegg, Germany) for 16 h at 4°C and was eluted from the beads, de-crosslinked, and DNA was extracted using the NucleoSpin® Gel and PCR Clean-up kit (Macherey-Nagel, Düren, Germany). The control (no-antibody) sample was produced by executing the same steps as mentioned earlier, but instead of GFP-Trap agarose beads, control agarose beads (i.e. without the immobilized anti-GFP antibody) were used for precipitation. Purified DNA samples were sequenced by Seqomics Ltd. (Mórahalom, Hungary).

The quality of reads was analyzed and filtered using fastqc.0.11.9. (https://www.bioinformatics.babraham.ac.uk/projects/fastqc/) and TrimmomaticPE.0.39. (Bolger et al. 2014) programs with the following parameters: TruSeq3-PE-2 adapters were cut from the reads with 2:30:10 values. The bases were cut from the end of a read if below 5 quality score. The reads were trimmed from the 5ʹ end if the average quality dropped <20 in a five-base-pair-long-sliding window. Reads with an average quality of <20 were dropped. The minimum length of the reads should be 50 base pairs. In the next step, the high-quality reads were mapped to *A. thaliana* reference genome (https://www.arabidopsis.org, TAIR10 chromosome files) using Bowtie.2.4.2. (Langmead and Salzberg 2012). PCR duplicates were identified with PicardTools MarkDuplicates.2.24.1. (http://broadinstitute.github.io/picard/). Sambamba.0.8.0. (Tarasov et al. 2015) was used to filter with the following parameter: ‘[XS] == null and not unmapped and not duplicate’. Additionally, properly paired reads were selected using Samtools.1.11. (Danecek et al. 2021) for further analysis. Reads that aligned to the mitochondrial and chloroplast genome were removed. In the next step, macs.2.2.7.1. (Zhang et al. 2008, Heinz et al. 2010) program was used to identify the significantly enriched peaks compared to no-antibody control (4-fold or higher enrichment). Peaks were called using the following parameters: 1.19e+8 genome size, 0.05 for minimum FDR cutoff and 147 bp extension size with ‘--no model’. In the next sept, the peaks were annotated using TAIR10 annotation file (https://www.arabidopsis.org, TAIR10 gff3) with HOMER’s annotatePeaks.pl program (Heinz et al. 2010). Peaks were assigned to genes with the nearest promoter. IGV genome bowser were used for data visualization (Robinson et al. 2011). **Supplementary Fig. S14** shows examples how the identified peaks were assigned to genes. Two biological replicates were processed for each genotype and condition. Genes represented by at least one peak in both replicates were considered binding targets.

Alternatively, a volume of 1.5 μl of the purified DNA was analyzed in qPCR reactions using iTaq Universal SYBR Green Supermix (Bio-Rad, Hercules, CA, USA,) according to the manufacturer’s recommendation. Short segments within genomic regions identified by ChIP-seq analysis were amplified. Standard series were prepared from 10-fold dilutions of the input DNA samples. The control (no antibody) sample was produced from PHYB-GFP/AbCDE samples by executing the same steps ChIP protocol as mentioned earlier, but instead of GFP-Trap agarose beads, control agarose beads (i.e. without the immobilized anti-GFP antibody) were used for precipitation. A control primer set specific to a genomic region between genes At4g26900 and At4g26910 served as non-binding probe/target. Enrichment was calculated by normalizing values to the signals measured in the initial non-immunoprecipitated (input) samples and then to the values obtained for the non-binding control region. According to this method of calculation, specific chromatin association is indicated by values >1. In case of ChIP-qPCR experiments, three biological replicates were processed for each genotype and condition.

### Transcriptomic analysis

Four-day-old seedlings were grown on half-strength MS plates at 17°C or 27°C in the dark as described earlier; 0.1 g plant material was snap frozen in liquid nitrogen from etiolated or 1 h or 24 h R (50 µmol m^−2^ s^−1^) illuminated seedlings. Total RNA was extracted using RNeasy Miniprep Kits (Qiagen, Germantown, MD, USA) according to the manufacturer’s instructions. RNA sequencing was done by Novogene, Cambridge, UK.

High-quality reads were achieved using fastqc.0.11.9 (https://www.bioinformatics.babraham.ac.uk/projects/fastqc/) and TrimmomaticPE.0.39. (Bolger et al. 2014), as described in the ChIP-seq analysis section. Next, the high-quality reads were mapped to *A. thaliana* reference genome (https://www.arabidopsis.org, TAIR10 chromosome files) using STAR.2.7.8a (Dobin et al. 2013) and sorted by coordinates using Samtools.1.11. (Danecek et al. 2021). Aligned reads were used to calculate the count data for all genes found in the *Arabidopsis* genome (https://www.arabidopsis.org, TAIR10 gff3) using htseq-count.0.13.5 (Anders et al. 2015). Next, tRNA, rRNA, mitochondrium and chloroplast genes were removed from the feature list. Genes having less than five counts per >75% of the samples were also excluded. Significant changes in gene expression elicited by light treatments were calculated from the log-transformed quantile-normalized intensities of the samples. For each temperature treatment, light exposure and genotype, plants kept in dark were used as dark control. The log2 fold changes and the FDR-adjusted *P*-values (*q*-values) were calculated using edgeR.3.38.4 (Huber et al. 2015; Robinson et al. 2010) library.

## Supplementary Material

pcae089_Supp

## Data Availability

ChIP-seq and transcriptome data can be accessed from the Gene Expression Omnibus repository (https://www.ncbi.nlm.nih.gov/geo/) with accession numbers GSE253043 and GSE253236.

## References

[R1] Ádám É., Kircher S., Liu P., Mérai Z., González-Schain N., Hörner M., et al. (2013) Comparative functional analysis of full-length and N-terminal fragments of phytochrome C, D and E in red light-induced signaling. *New Phytol*. 200: 86–96. 23772959 10.1111/nph.12364

[R2] Anders S., Pyl P.T. and Huber W. (2015) HTSeq—a Python framework to work with high-throughput sequencing data. *Bioinformatics (Oxford, England)* 31: 166–169. 25260700 10.1093/bioinformatics/btu638 PMC4287950

[R3] Aukerman M.J., Hirschfeld M., Wester L., Weaver M., Clack T., Amasino R.M., et al. (1997) A deletion in the PHYD gene of the *Arabidopsis* Wassilewskija ecotype defines a role for phytochrome D in red/far-red light sensing. *Plant Cell* 9: 1317–1326. 9286109 10.1105/tpc.9.8.1317 PMC157000

[R4] Bae G. and Choi G. (2008) Decoding of light signals by plant phytochromes and their interacting proteins. *Annu. Rev. Plant Biol*. 59: 281–311. 18257712 10.1146/annurev.arplant.59.032607.092859

[R5] Bolger A.M., Lohse M. and Usadel B. (2014) Trimmomatic: a flexible trimmer for Illumina sequence data. *Bioinformatics (Oxford, England)* 30: 2114–2120. 24695404 10.1093/bioinformatics/btu170 PMC4103590

[R6] Briggs W.R. and Christie J.M. (2002) Phototropins 1 and 2: versatile plant blue-light receptors. *Trends Plant Sci*. 7: 204–210. 11992825 10.1016/s1360-1385(02)02245-8

[R7] Burko Y., Seluzicki A., Zander M., Pedmale U.V., Ecker J.R. and Chory J. (2020) Chimeric activators and repressors define HY5 activity and reveal a light-regulated feedback mechanism. *Plant Cell* 32: 967–983. 32086365 10.1105/tpc.19.00772 PMC7145465

[R8] Chen F., Li B., Li G., Charron J.-B., Dai M., Shi X., et al. (2014) *Arabidopsis* phytochrome a directly targets numerous promoters for individualized modulation of genes in a wide range of pathways. *Plant Cell* 26: 1949–1966. 24794133 10.1105/tpc.114.123950 PMC4079361

[R9] Clack T., Shokry A., Moffet M., Liu P., Faul M. and Sharrock R.A. (2009) Obligate heterodimerization of *Arabidopsis* phytochromes C and E and interaction with the PIF3 basic helix-loop-helix transcription factor. *Plant Cell* 21: 786–799. 19286967 10.1105/tpc.108.065227 PMC2671712

[R10] Clough S.J. and Bent A.F. (1998) Floral dip: a simplified method for Agrobacterium-mediated transformation of *Arabidopsis thaliana*. *Plant J*. 16: 735–743. 10069079 10.1046/j.1365-313x.1998.00343.x

[R11] Danecek P., Bonfield J.K., Liddle J., Marshall J., Ohan V., Pollard M.O., et al. (2021) Twelve years of SAMtools and BCFtools. *GigaScience* 10: giab008. 10.1093/gigascience/giab008 PMC7931819 33590861

[R12] Demarsy E. and Fankhauser C. (2009) Higher plants use LOV to perceive blue light. *Curr. Opin. Plant Biol*. 12: 69–74. 18930433 10.1016/j.pbi.2008.09.002

[R13] Devlin P.F., Patel S.R. and Whitelam G.C. (1998) Phytochrome E influences internode elongation and flowering time in *Arabidopsis*. *Plant Cell* 10: 1479–1487. 9724694 10.1105/tpc.10.9.1479 PMC144080

[R14] Devlin P.F., Robson P.R., Patel S.R., Goosey L., Sharrock R.A. and Whitelam G.C. (1999) Phytochrome D acts in the shade-avoidance syndrome in *Arabidopsis* by controlling elongation growth and flowering time. *Plant Physiol*. 119: 909–915. 10069829 10.1104/pp.119.3.909 PMC32105

[R15] Dobin A., Davis C.A., Schlesinger F., Drenkow J., Zaleski C., Jha S., et al. (2013) STAR: ultrafast universal RNA-seq aligner. *Bioinformatics (Oxford, England)* 29: 15–21. 23104886 10.1093/bioinformatics/bts635 PMC3530905

[R16] Dobos O., Horvath P., Nagy F., Danka T. and Viczián A. (2019) A deep learning-based approach for high-throughput hypocotyl phenotyping. *Plant Physiol*. 181: 1415–1424. 31636105 10.1104/pp.19.00728 PMC6878028

[R17] Fankhauser C. and Chen M. (2008) Transposing phytochrome into the nucleus. *Trends Plant Sci*. 13: 596–601. 18824397 10.1016/j.tplants.2008.08.007

[R18] Fernández A.P., Gil P., Valkai I., Nagy F. and Schäfer E. (2005) Analysis of the function of the photoreceptors phytochrome B and phytochrome D in *Nicotiana plumbaginifolia* and *Arabidopsis thaliana*. *Plant Cell Physiol*. 46: 790–796. 15753105 10.1093/pcp/pci073

[R19] Franklin K.A., Praekelt U., Stoddart W.M., Billingham O.E., Halliday K.J. and Whitelam G.C. (2003) Phytochromes B, D, and E act redundantly to control multiple physiological responses in *Arabidopsis*. *Plant Physiol*. 131: 1340–1346. 12644683 10.1104/pp.102.015487 PMC166893

[R20] González-Grandío E., Álamos S., Zhang Y., Dalton-Roesler J., Niyogi K.K., García H.G., et al. (2022) Chromatin changes in phytochrome interacting factor-regulated genes parallel their rapid transcriptional response to light. *Front. Plant Sci*. 13: 803441. 10.3389/fpls.2022.803441 PMC8891703 35251080

[R21] Halliday K.J. and Whitelam G.C. (2003) Changes in photoperiod or temperature alter the functional relationships between phytochromes and reveal roles for phyD and phyE. *Plant Physiol*. 131: 1913–1920. 12692350 10.1104/pp.102.018135 PMC166947

[R22] Heinz S., Benner C., Spann N., Bertolino E., Lin Y.C., Laslo P., et al. (2010) Simple combinations of lineage-determining transcription factors prime cis-regulatory elements required for macrophage and B cell identities. *Mol. Cell* 38: 576–589. 20513432 10.1016/j.molcel.2010.05.004 PMC2898526

[R23] Hu W., Franklin K.A., Sharrock R.A., Jones M.A., Harmer S.L. and Lagarias J.C. (2013) Unanticipated regulatory roles for *Arabidopsis* phytochromes revealed by null mutant analysis. *Proc. Natl. Acad. Sci. U.S.A*. 110: 1542–1547. 23302690 10.1073/pnas.1221738110 PMC3557068

[R24] Huber W., Carey V.J., Gentleman R., Anders S., Carlson M., Carvalho B.S., et al. (2015) Orchestrating high-throughput genomic analysis with bioconductor. *Nat. Methods* 12: 115–121. 25633503 10.1038/nmeth.3252 PMC4509590

[R25] Jung J.-H., Domijan M., Klose C., Biswas S., Ezer D., Gao M., et al. (2016) Phytochromes function as thermosensors in *Arabidopsis*. *Science (New York, N.Y.)* 354: 886–889. 27789797 10.1126/science.aaf6005

[R26] Kerbler S.M. and Wigge P.A. (2023) Temperature sensing in plants. *Annu. Rev. Plant Biol*. 74: 341–366. 36854477 10.1146/annurev-arplant-102820-102235

[R27] Kim C., Kwon Y., Jeong J., Kang M., Lee G.S., Moon J.H., et al. (2023) Phytochrome B photobodies are comprised of phytochrome B and its primary and secondary interacting proteins. *Nat. Commun*. 14: 1708. 10.1038/s41467-023-37421-z PMC10042835 36973259

[R28] Kircher S., Gil P., Kozma-Bognár L., Fejes E., Speth V., Husselstein-Muller T., et al. (2002) Nucleocytoplasmic partitioning of the plant photoreceptors phytochrome A, B, C, D, and E is regulated differentially by light and exhibits a diurnal rhythm. *Plant Cell* 14: 1541–1555. 12119373 10.1105/tpc.001156 PMC150705

[R29] Klose C. (2019) In vivo spectroscopy. *Methods Mol. Biol*. 2026: 113–120. 31317406 10.1007/978-1-4939-9612-4_8

[R30] Klose C., Nagy F. and Schäfer E. (2020) Thermal reversion of plant phytochromes. *Mol. Plant* 13: 386–397. 31812690 10.1016/j.molp.2019.12.004

[R31] Klose C., Viczián A., Kircher S., Schäfer E. and Nagy F. (2015) Molecular mechanisms for mediating light-dependent nucleo/cytoplasmic partitioning of phytochrome photoreceptors. *New Phytol*. 206: 965–971. 26042244 10.1111/nph.13207 PMC4406131

[R32] Langmead B. and Salzberg S.L. (2012) Fast gapped-read alignment with Bowtie 2. *Nat. Methods* 9: 357–359. 22388286 10.1038/nmeth.1923 PMC3322381

[R33] Legris M., Klose C., Burgie E.S., Rojas C.C.R., Neme M., Hiltbrunner A., et al. (2016) Phytochrome B integrates light and temperature signals in *Arabidopsis*. *Science (New York, N.Y.)* 354: 897–900. 27789798 10.1126/science.aaf5656

[R34] Leivar P., Monte E., Oka Y., Liu T., Carle C., Castillon A., et al. (2008) Multiple phytochrome-interacting bHLH transcription factors repress premature seedling photomorphogenesis in darkness. *Curr. Biol*. 18: 1815–1823. 19062289 10.1016/j.cub.2008.10.058 PMC2651225

[R35] Liu P. and Sharrock R.A. (2013) Directed dimerization: an in vivo expression system for functional studies of type II phytochromes. *Plant J*. 75: 915–926. 23738620 10.1111/tpj.12255

[R36] Mathews S. (2005) Phytochrome evolution in green and nongreen plants. *J. Hered*. 96: 197–204. 15695552 10.1093/jhered/esi032

[R37] Mathews S. and McBreen K. (2008) Phylogenetic relationships of B-related phytochromes in the Brassicaceae: redundancy and the persistence of phytochrome D. *Mol. Phylogen. Evol*. 49: 411–423. 10.1016/j.ympev.2008.07.026 18768161

[R38] Medzihradszky M., Bindics J., Ádám É., Viczián A., Klement É., Lorrain S., et al. (2013) Phosphorylation of phytochrome B inhibits light-induced signaling via accelerated dark reversion in *Arabidopsis*. *Plant Cell* 25: 535–544. 23378619 10.1105/tpc.112.106898 PMC3608776

[R39] Monte E., Alonso J.M., Ecker J.R., Zhang Y., Li X., Young J., et al. (2003) Isolation and characterization of phyC mutants in *Arabidopsis* reveals complex crosstalk between phytochrome signaling pathways. *Plant Cell* 15: 1962–1980. 12953104 10.1105/tpc.012971 PMC181324

[R40] Nagy F. and Schäfer E. (2002) Phytochromes control photomorphogenesis by differentially regulated, interacting signaling pathways in higher plants. *Annu. Rev. Plant Biol*. 53: 329–355. 12221979 10.1146/annurev.arplant.53.100301.135302

[R41] Ni W., Xu S.-L., Tepperman J.M., Stanley D.J., Maltby D.A., Gross J.D., et al. (2014) A mutually assured destruction mechanism attenuates light signaling in *Arabidopsis*. *Science (New York, N.Y.)* 344: 1160–1164. 24904166 10.1126/science.1250778 PMC4414656

[R42] Quail P.H. (2002) Phytochrome photosensory signalling networks. *Nat. Rev. Mol. Cell Biol*. 3: 85–93. 11836510 10.1038/nrm728

[R43] Rizzini L., Favory -J.-J., Cloix C., Faggionato D., O’Hara A., Kaiserli E., et al. (2011) Perception of UV-B by the *Arabidopsis* UVR8 protein. *Science (New York, N.Y.)* 332: 103–106. 21454788 10.1126/science.1200660

[R44] Robinson J.T., Thorvaldsdóttir H., Winckler W., Guttman M., Lander E.S., Getz G., et al. (2011) Integrative genomics viewer. *Nat. Biotechnol*. 29: 24–26. 21221095 10.1038/nbt.1754 PMC3346182

[R45] Robinson M.D., McCarthy D.J. and Smyth G.K. (2010) edgeR: a Bioconductor package for differential expression analysis of digital gene expression data. *Bioinformatics (Oxford, England)* 26: 139–140. 19910308 10.1093/bioinformatics/btp616 PMC2796818

[R46] Rockwell N.C., Su Y.-S. and Lagarias J.C. (2006) Phytochrome structure and signaling mechanisms. *Annu. Rev. Plant Biol*. 57: 837–858. 16669784 10.1146/annurev.arplant.56.032604.144208 PMC2664748

[R47] Sánchez-Lamas M., Lorenzo C.D., Cerdán P.D. and Fankhauser C. (2016) Bottom-up assembly of the phytochrome network. *PLoS Genet*. 12: e1006413. 10.1371/journal.pgen.1006413 PMC5098793 27820825

[R48] Sharrock R.A. and Clack T. (2004) Heterodimerization of type II phytochromes in *Arabidopsis*. *Proc. Natl. Acad. Sci. U.S.A*. 101: 11500–11505. 15273290 10.1073/pnas.0404286101 PMC509229

[R49] Sharrock R.A., Clack T. and Goosey L. (2003a) Differential activities of the *Arabidopsis* phyB/D/E phytochromes in complementing phyB mutant phenotypes. *Plant Mol. Biol*. 52: 135–142. 12825695 10.1023/a:1023901718508

[R50] Sharrock R.A., Clack T. and Goosey L. (2003b) Signaling activities among the *Arabidopsis* phyB/D/E-type phytochromes: a major role for the central region of the apoprotein. *Plant J*. 34: 317–326. 12713538 10.1046/j.1365-313x.2003.01722.x

[R51] Shin J., Kim K., Kang H., Zulfugarov I.S., Bae G., Lee C.-H., et al. (2009) Phytochromes promote seedling light responses by inhibiting four negatively-acting phytochrome-interacting factors. *Proc. Natl. Acad. Sci. U.S.A*. 106: 7660–7665. 19380720 10.1073/pnas.0812219106 PMC2678665

[R52] Strasser B., Sánchez-Lamas M., Yanovsky M.J., Casal J.J. and Cerdán P.D. (2010) *Arabidopsis thaliana* life without phytochromes. *Proc. Natl. Acad. Sci. U.S.A*. 107: 4776–4781. 20176939 10.1073/pnas.0910446107 PMC2842051

[R53] Sun Y., Fan X.-Y., Cao D.-M., Tang W., He K., Zhu J.-Y., et al. (2010) Integration of brassinosteroid signal transduction with the transcription network for plant growth regulation in *Arabidopsis*. *Dev. Cell* 19: 765–777. 21074725 10.1016/j.devcel.2010.10.010 PMC3018842

[R54] Tarasov A., Vilella A.J., Cuppen E., Nijman I.J. and Prins P. (2015) Sambamba: fast processing of NGS alignment formats. *Bioinformatics (Oxford, England)* 31: 2032–2034. 25697820 10.1093/bioinformatics/btv098 PMC4765878

[R55] Vanhaelewyn L., Bernula P., Van Der Straeten D., Vandenbussche F. and Viczián A. (2019) UVR8-dependent reporters reveal spatial characteristics of signal spreading in plant tissues. *Photochem. Photobiol. Sci*. 18: 1030–1045. 30838366 10.1039/c8pp00492g

[R56] Viczián A., Ádám É., Staudt A.-M., Lambert D., Klement E., Romero Montepaone S., et al. (2020) Differential phosphorylation of the N-terminal extension regulates phytochrome B signaling. *New Phytol*. 225: 1635–1650. 31596952 10.1111/nph.16243

[R57] Wang Q. and Lin C. (2020) Mechanisms of cryptochrome-mediated photoresponses in plants. *Annu. Rev. Plant Biol*. 71: 103–129. 32169020 10.1146/annurev-arplant-050718-100300 PMC7428154

[R58] Wang Z.-Y., Wang Q., Chong K., Wang F., Wang L., Bai M., et al. (2006) The brassinosteroid signal transduction pathway. *Cell Res*. 16: 427–434. 16699538 10.1038/sj.cr.7310054 PMC2990686

[R59] Wu J., Wang W., Xu P., Pan J., Zhang T., Li Y., et al. (2019) phyB interacts with BES1 to regulate brassinosteroid signaling in *Arabidopsis*. *Plant Cell Physiol*. 60: 353–366. 30388258 10.1093/pcp/pcy212

[R60] Yu X., Li L., Zola J., Aluru M., Ye H., Foudree A., et al. (2011) A brassinosteroid transcriptional network revealed by genome-wide identification of BESI target genes in *Arabidopsis thaliana*. *Plant J*. 65: 634–646. 21214652 10.1111/j.1365-313X.2010.04449.x

[R61] Zhang Y., Liu T., Meyer C.A., Eeckhoute J., Johnson D.S., Bernstein B.E., et al. (2008) Model-based analysis of ChIP-Seq (MACS). *Genome Biol*. 9: R137. 10.1186/gb-2008-9-9-r137 PMC2592715 18798982

