# Supplementary material for: Phytochrome C and Low Temperature Promote the Protein Accumulation and Red-Light Signaling of Phytochrome D
Source: Plant Cell Physiol. 2024 Aug 9;65(10):1717–35. doi: 10.1093/pcp/pcae089 (PMC11558544; doi:10.1093/pcp/pcae089)
Supplement: pcae089_Supp [file pcae089_supp.zip › suppl_data/pcp-2024-e-00164-File010.pdf]

Phytochrome C and Low Temperature Promote the Protein Accumulation and Red Light Signaling of Phytochrome D

Csaba Péter, Éva Ádám, Cornelia Klose, Gábor Grézal, Anita Hajdu, Gábor Steinbach, László Kozma-Bognár, Dániel Silhavy, Ferenc Nagy and András Viczián

SUPPLEMENTARY TABLES AND FIGURES

	PHYD-YFP/abcde				PHYD-YFP/abCde				aBcde			
	17 °C		27 °C		17 °C		27 °C		17 °C		27 °C	
	1 h	24 h	1 h	24 h	1 h	24 h	1 h	24 h	1 h	24 h	1 h	24 h
**up**	1	501	6	272	3	996	9	1108	559	1906	106	1690
**down**	6	183	4	41	4	738	21	704	898	1678	136	1038
**total**	7	684	10	313	7	1734	30	1812	1457	3584	242	2728

Supplementary Table S1. Differentially expressed genes (DEGs).
RNA-seq was conducted from PHYD-YFP/abcde, PHYD-YFP/abCde and aBcde lines grown in dark at 17 or 27 °C, and then the plants were treated with red light for 1 or 24 hours (h). Categories named ‚up’ and ‚down’ show the number of genes that showed significant (adjusted p value <0.05) and at least two times up- or downregulation relative to the corresponding dark control. Category named ‚total’ contains the summary of ‚up’ and ‚down’.

	PHYD-YFP/abcde	PHYD-YFP/abCde	PHYB-GFP/AbCDE
Total number of peaks (the sum of the number of peaks in all replicates)	49	1006	136
Number of unique genes associated with at least 1 peak at least in one replicate sample	39	880	67
Number of unique genes associated with 1 peak at least in one replicate sample	37	840	54
Number of unique genes associated with 2 peaks at least in one replicate sample	2	35	8
Number of unique genes associated with 3 peaks at least in one replicate sample	0	5	3
Number of unique genes associated with 4 peaks at least in one replicate sample	0	0	2
**Number of unique genes associated with at least 1 peak in two replicate samples**	**8**	**74**	**38**

Supplementary Table S2. Statistics on the association of peaks identified by the ChIP-seq assay with genes.
Based on the data shown in Supplementary dataset S4, the number of all peaks and the corresponding genes were calculated and are listed here.

**YFP_RT Fwd**	TTTACCAGACAACCATTACCTG
**YFP_RT Rev**	GTTACAAACTCAAGAAGGACCA
**PHYD_RT Fw**	GACATGGATTTAAACGAGGATCAG
**PHYD_RT Rev**	TCTCTAGCAGAAATGAACCGT
**TUB2/3-RT Fwd**	CCAGCTTTGGTGATTTGAAC
**TUB2/3_RT Rev**	CAAGCTTTCGGAGGTCAGAG
**At4g26900/10 ChIP Fwd**	TCTTATAGTTGATTTCCTTTTGTTGACAGT
**At4g26900/10 ChIP Rev**	GCTGAGAAAGTGAACATACGTTGCT
**PIL1 ChIP Fwd**	ATAACACAAAGGGGTGGATG
**PIL1 ChIP Rev**	TAAATGGGACCCACAATTAG
**BBX28 ChIP Fwd**	GCCCATTAAACTTGTGTTATGGTC
**BBX28 ChIP Rev**	AGGAGGAGAGCGAGATTCTG
**BGH2 ChIP Fwd**	GTAGTGTGGGTCCTACTTCA
**BGH2 ChIP Rev**	TATGCTTATGATTGGCTGAGAG
**BBX29 ChIP Fwd**	AGACGTGTATTGAGGACCAG
**BBX29 ChIP Rev**	TCGTATAGAGGAGAGGGAAGAT
**ATHB2 ChIP Fwd**	GTAGTGTTCACGTGCATGTC
**ATHB2 ChIP Rev**	GCTAATCGACAAGTCAACATCC
**PIL2 ChIP Fwd**	GTTTCACAAACCGATAGTACGAC
**PIL2 ChIP Rev**	TGAATGTTCTTCCCACAACCA

Supplementary Table S3. Primers used in the study.

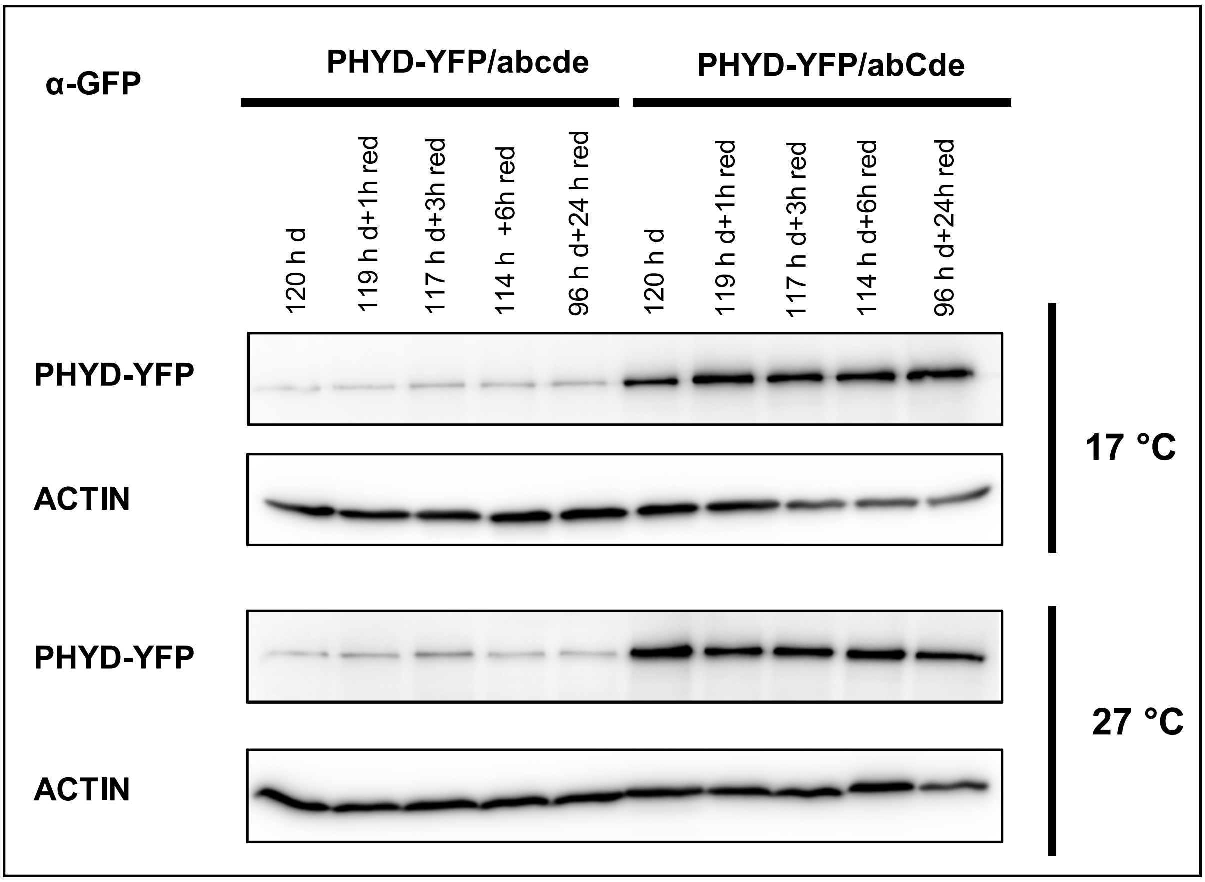

Supplementary Fig. S1. Short irradiation of red light does not modify PHYD-YFP accumulation.

Seedlings expressing *35S:PHYD-YFP* either in the abcde or in the abCde genetic background were grown in the dark (d), irradiated with constant red light (50 µmol m^{-2} s^{-1}) for the indicated times at 17 or at 27 °C. The amount of PHYD-YFP protein was determined by immunoblotting using anti-GFP antibody. ACTIN was used as loading control.

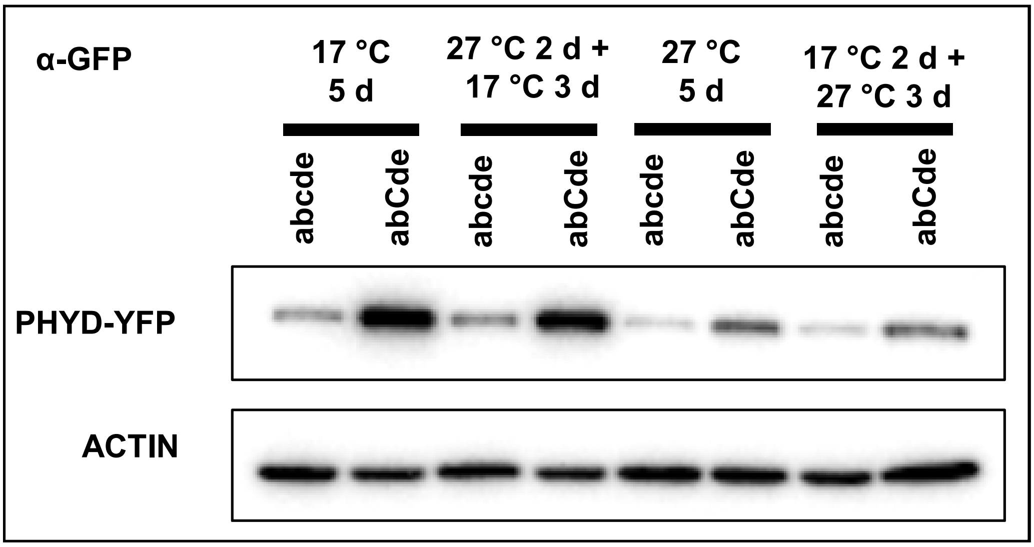

Supplementary Fig. S2. Temperature shift modifies PHYD-YFP protein accumulation.
Seedlings expressing *35S:PHYD-YFP* either in the abcde or in the abCde genetic background were grown under constant R light (50 µmol m^{-2} s^{-1}) irradiation either at 17 or at 27 °C for 5 days or 2 days and shifted to the other temperature (17-to-27 or 27-to-17 °C) for 3 days. The amount of PHYD-YFP protein was determined by immunoblotting using anti-GFP antibody. ACTIN was used as loading control.

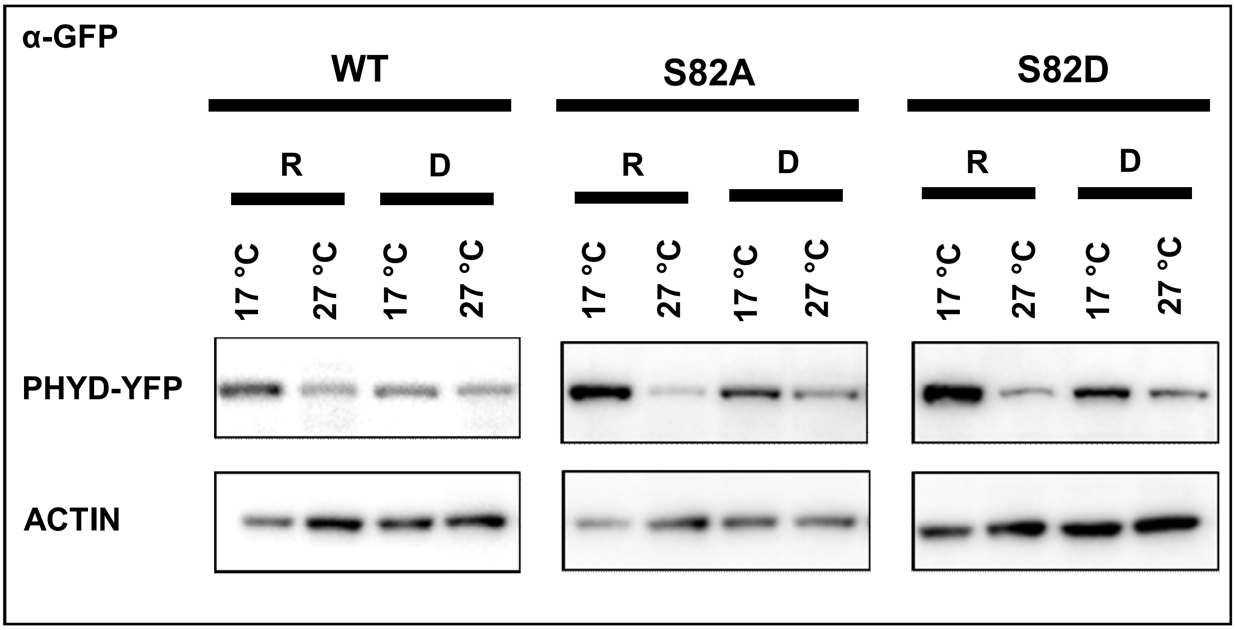

Figure S3. Accumulation of PHYD-YFP protein depends on the temperature in the dark and under constant R irradiation.
Seedlings expressing *35S:PHYD-YFP* (WT) or its mutant versions containing the serine 82 substitution to alanine (S82A) or aspartic acid (S82D) in the abCdE genetic background were grown in the dark (D) or under constant red irradiation (R, 50 µmol m^{-2} s^{-1}) for 5 days at 17 or 27 °C. Amounts of PHYD-YFP protein was determined by immunoblotting using anti-GFP antibody and ACTIN is used as loading control.

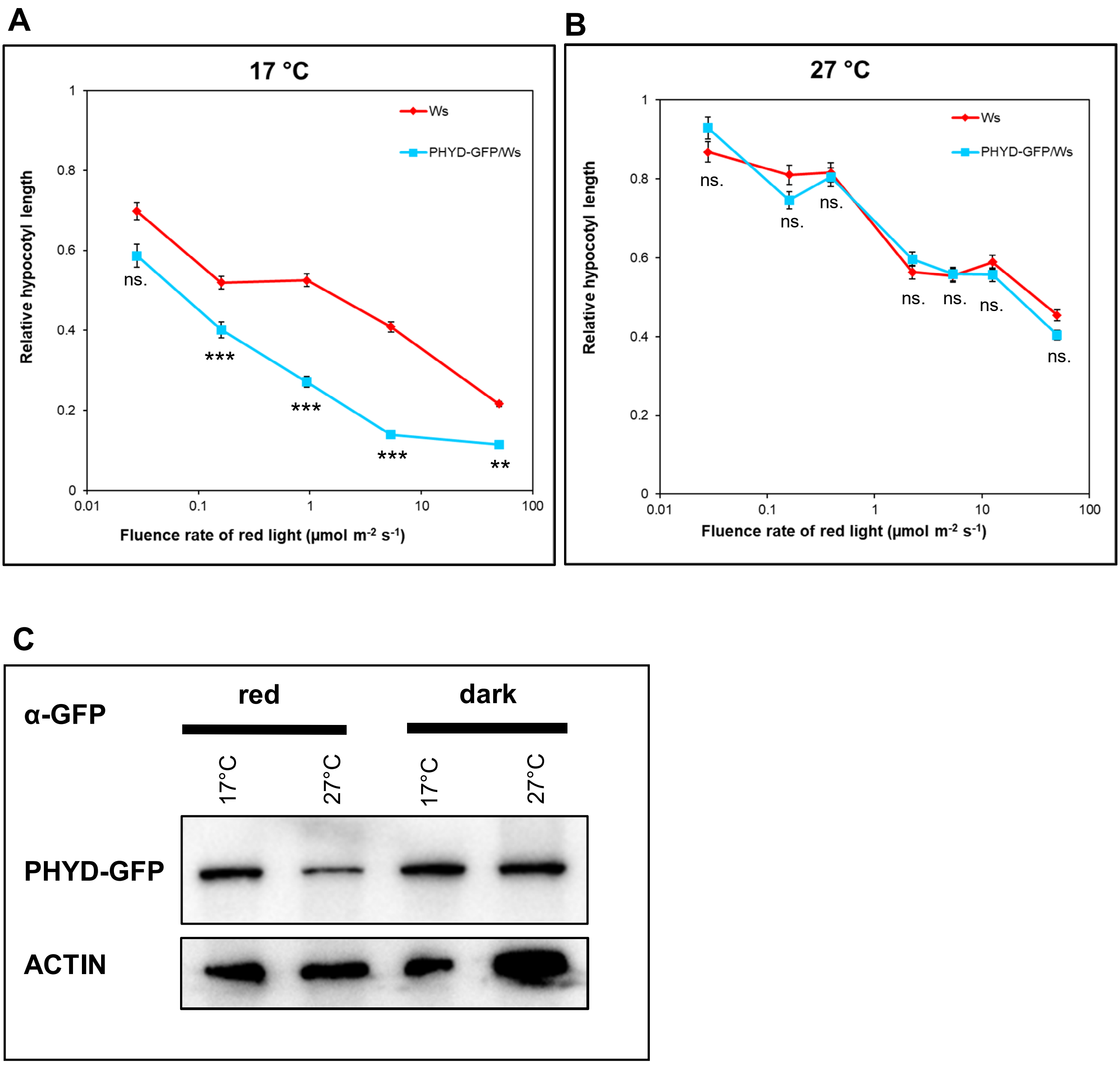

Supplementary Fig. S4. PHYD-GFP effectively modulates photomorphogenesis in Ws ecotype at lower temperature.

Seedlings were grown at either 17 °C (A) or at 27 °C (B) for 4 days under different fluences of constant red light irradiation. Hypocotyl values relative to the corresponding dark controls are shown. n≥30, error bars indicate standard errors. Asterisks denote significant difference between the 17 and 27 °C treatment (Mann-Whitney U test, ** p<0.01; *** p<0.001; ns.: not significant). (C) PHYD-YFP/Ws seedlings were grown as in (A) and (B) and the amount of PHYD-GFP protein was determined by immunoblotting using anti-GFP antibody. ACTIN was used as loading control.

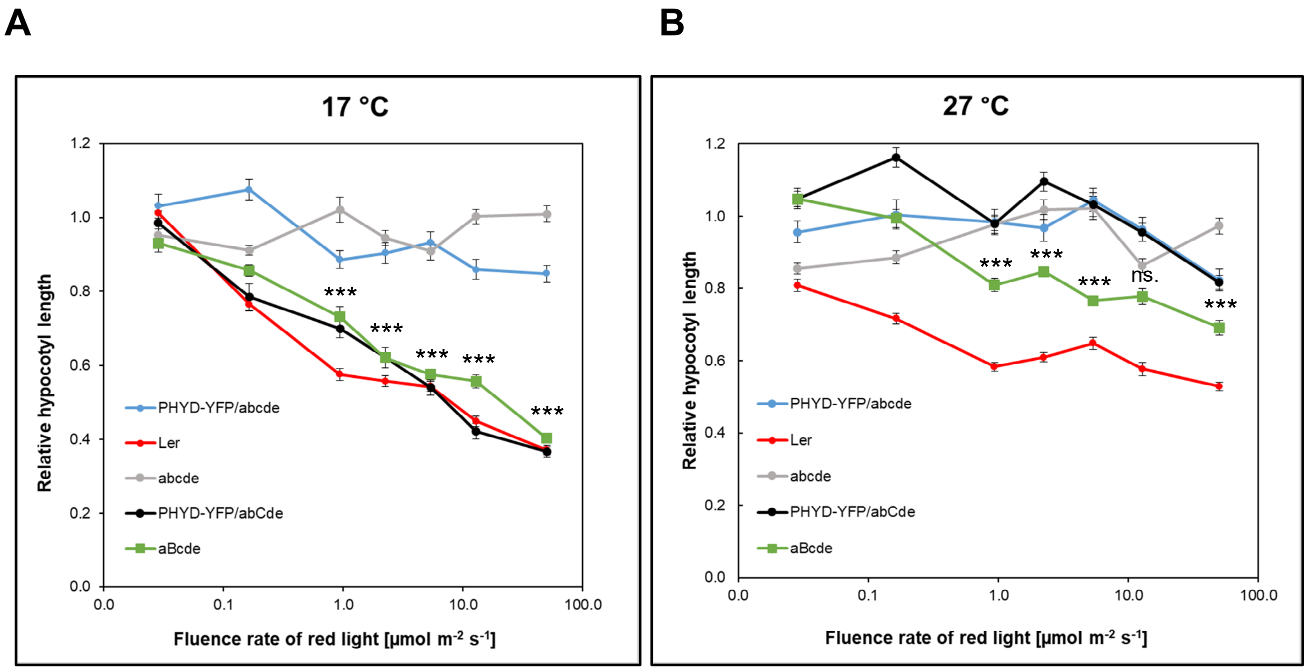

Supplementary Fig. S5. phyB mediates efficient red light signaling both at 17 and 27 °C. Seedlings were grown at either 17 °C (A) or at 27 °C (B) for 4 days under different fluences of constant R light irradiation. Hypocotyl values relative to the corresponding dark controls are shown. n≥30, error bars indicate standard errors. Asterisks denote significant difference between the aBcde and the abcde lines at the marked data points (Mann-Whitney U test, *** *p*<0.001; ns.: not significant). Data are taken from Fig. 1, supplemented with the values of the aBcde line.

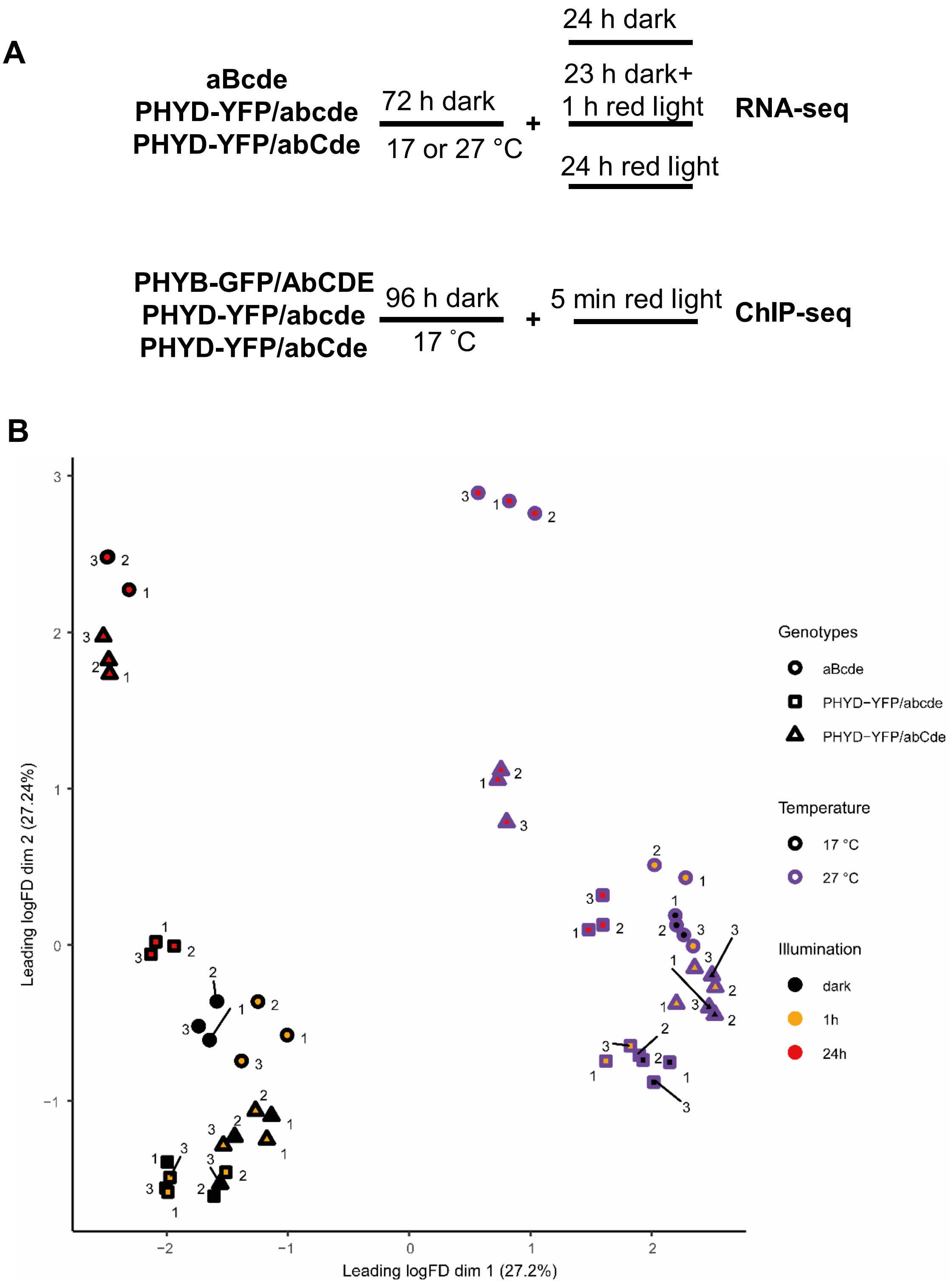

Supplementary Fig. S6. Effect of red light treatment on gene expression in different *Arabidopsis* phytochrome mutants.

(A) Schematic representation of the experimental setup for comparative RNA- and ChIP-seq analysis. (B) PCA analyses of transcriptomes of dark-grown and red light treated samples. RNA-seq was conducted from samples (three repeats) isolated from PHYD-YFP/abcde, PHYD-YFP/abCde and aBcde Arabidopsis lines grown at 17 or 27 °C in dark for 96 h or dark-grown seedlings were red light treated for 1 h or for 24 h. Note that the repeats of all samples are well clustered.

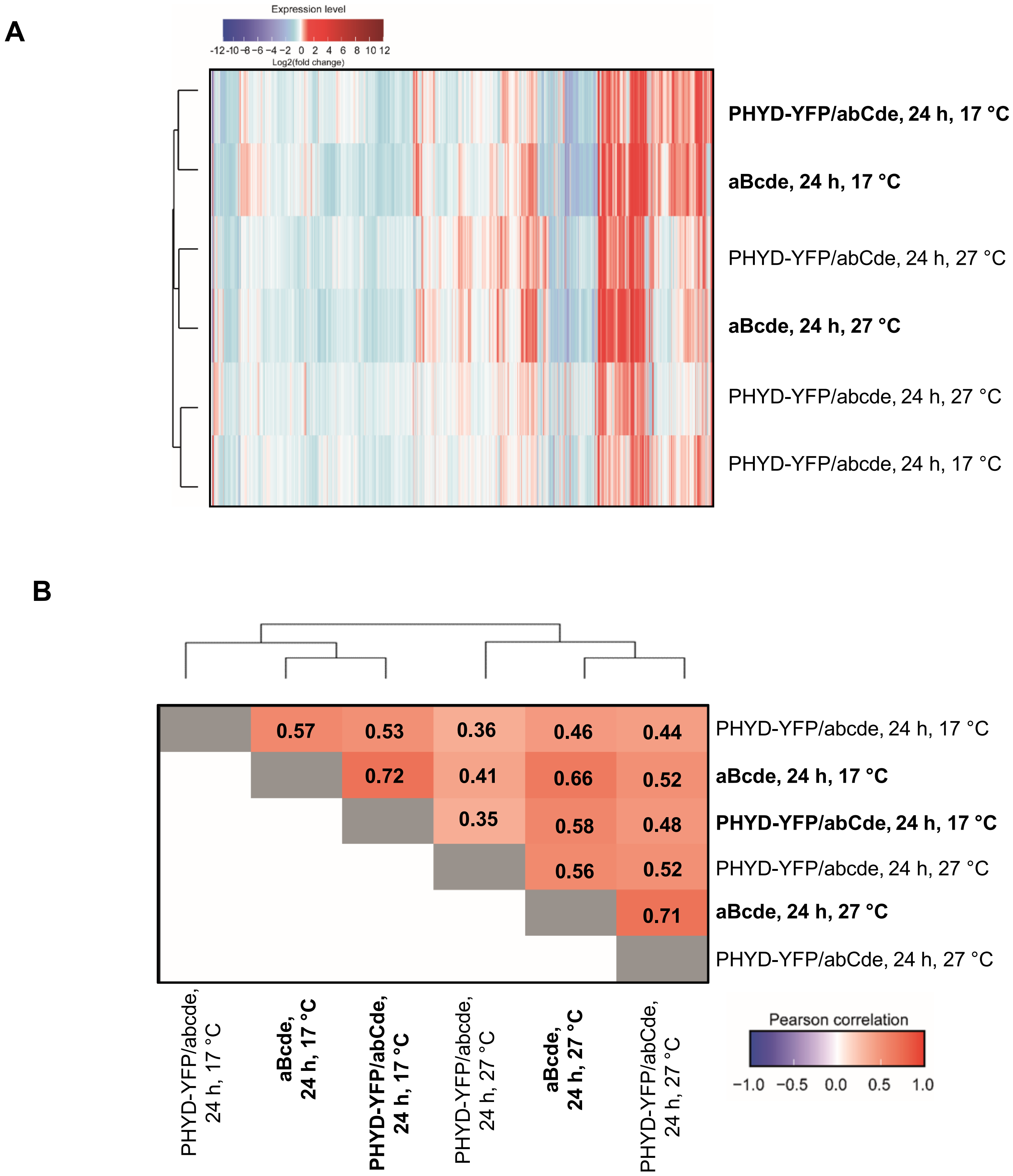

Supplementary Fig. S7. 24 h long red light illumination induced transcriptional changes in different Arabidopsis phytochrome mutants.
(A) Heat-map of transcriptome changes in response to 24 h red light treatment. .(B) Pearson correlation of transcriptome changes after 24 h red light treatment. Plants that showed the photomorphogenesis response of hypocotyl shortening at the given condition are printed in bold

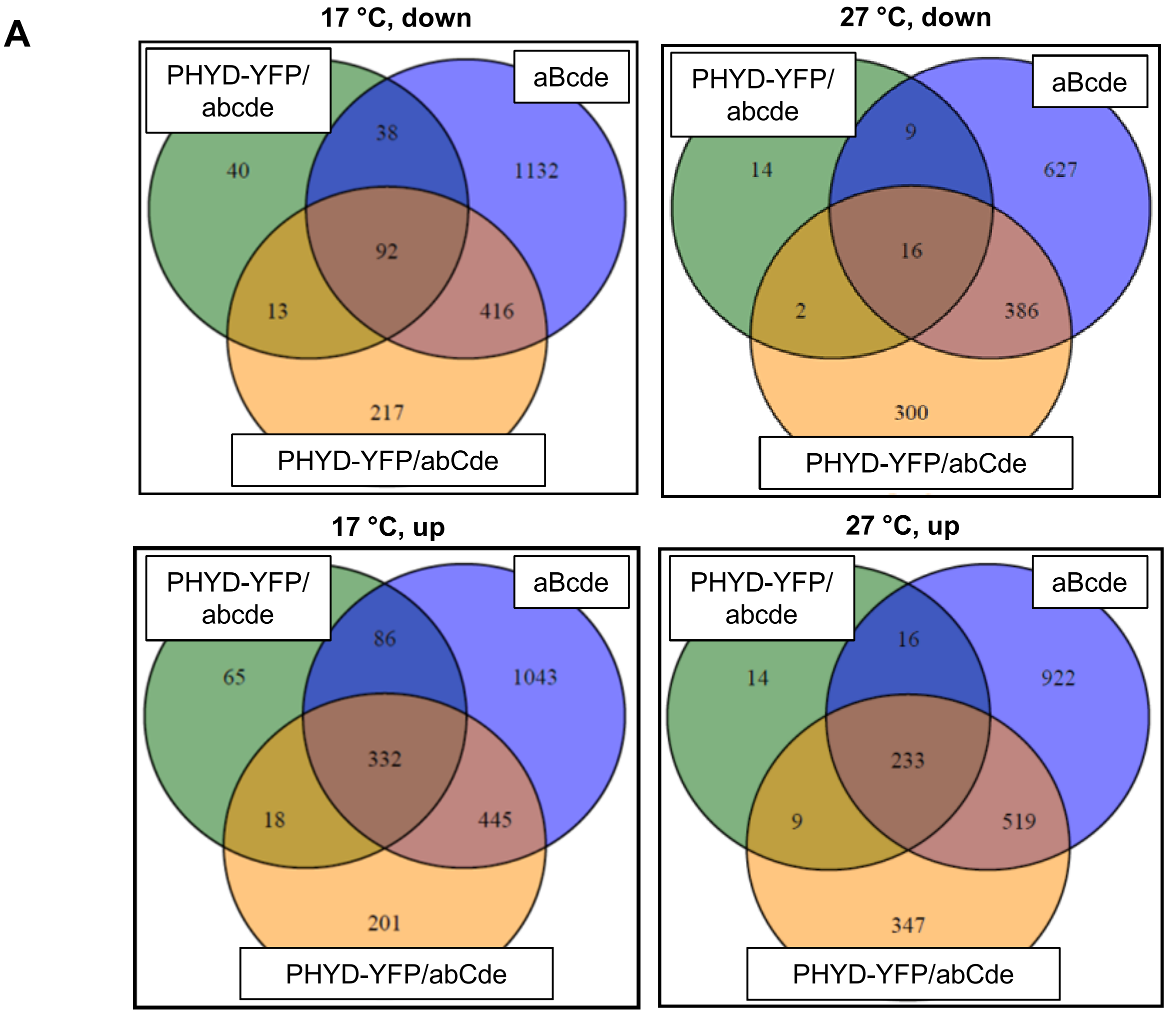

B

**PHYD-YFP/abCde and aBcde Overlapping DEGs**	**17 °C**	**27 °C**
**Up**	777/996<br>**78% *****	752/1108<br>**67%*****
**Down**	508/738<br>**68%*****	402/704<br>**57%*****
**Total**	1285/1734<br>**74%*****	1154/1812<br>**63%*****

Supplementary Fig. S8. Differentially Expressed Genes (DEGs) after 24 h light treatment in different phytochrome mutants.
(A) Venn diagrams of Differentially Expressed Genes (DEGs). (B) Overlapping DEGs of PHYD-YFP/abCde and aBcde plants. The number of DEGs, which are significantly modified in the same direction in both PHYD-YFP/abCde and aBcde plants, was divided by the total number of DEGs (modified in the same direction) in PHYD-YFP/abCde plants. Note that most of the PHYD-YFP/abCde DEGs are regulated similarly in aBcde, especially at low temperature. Statistical evaluation of data suggested significantly greater overlap of the indicated DEGs than it had been expected based on random distribution. *** marks significance at $p<0.000001$ as calculated by Fisher's Exact Test.

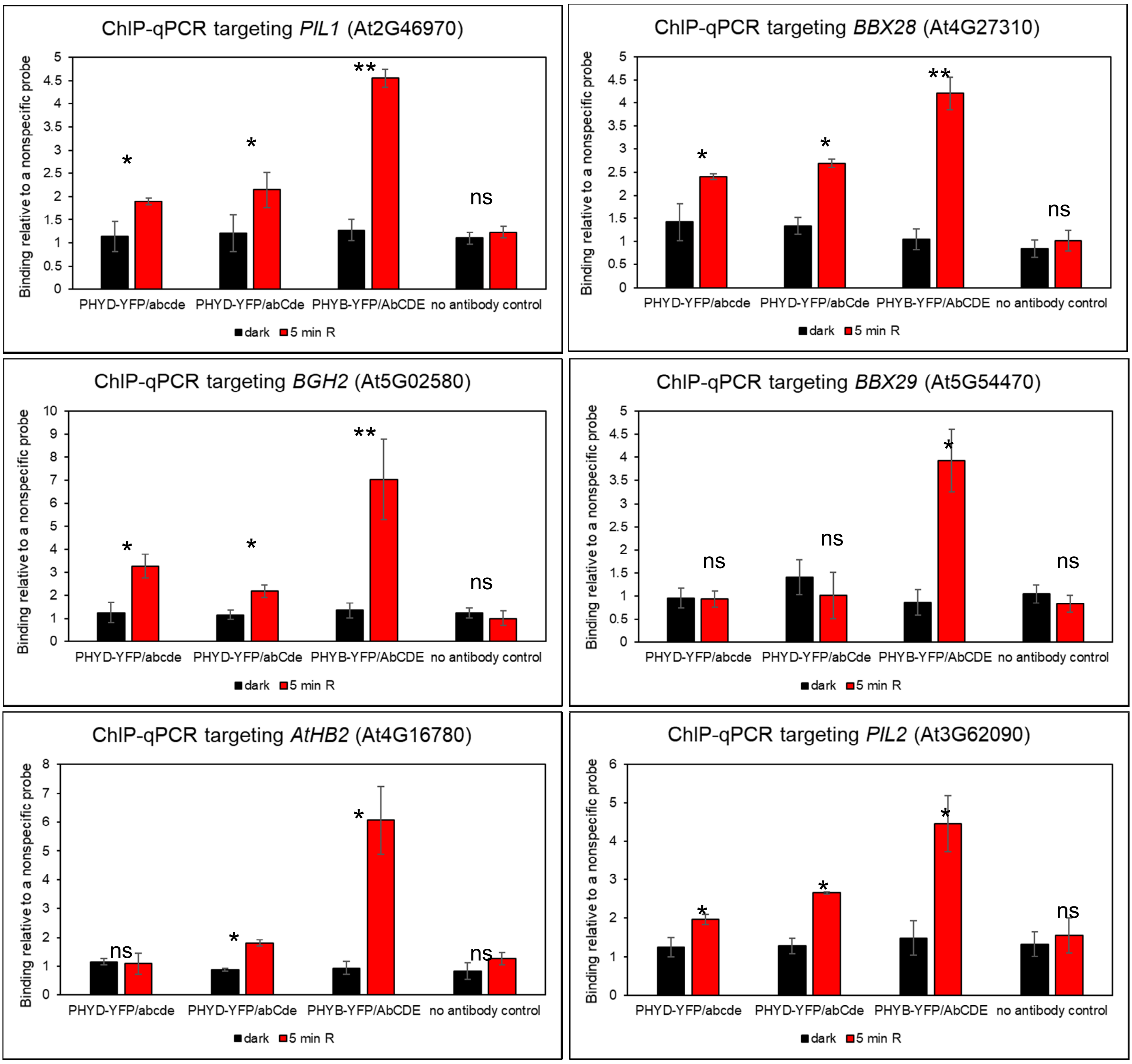

Supplementary Fig. S9. Light-dependent chromatin association of phyD-YFP and phyB-GFP. PHYD-YFP/abcde, PHYD-YFP/abCde and PHYB-GFP/AbCDE plants were grown in darkness for 6 days at 17 °C. Plants were irradiated with red light (50 µmol m^{-2} s^{-1}) 5 min (5 min R) or kept in darkness for additional 5 min (dark) before fixing in 1% (v/v) formaldehyde solution. Chromatin precipitation and purification of DNA fragments were done as for the ChIP-seq analysis previously. The no antibody control was obtained from ChIP performed on PHYB-GFP/AbCDE samples without antibody. Specific DNA fragments were detected and quantified by qRT-PCR. Primers were designed to amplify fragments that were identified in previous ChIP-seq assays as regions bound by phyD-YFP and phyB-GFP, and were recovered from all ChIP-seq samples (for examples see Fig. S14). Values were first normalized to the values of the initial non-immunoprecipitated input DNA samples and then to the values obtained for a non-bound intergenic fragment located between At4g26900 and At4g26910. In this way, values above 1 indicate chromatin binding. Three independent biological replicates were processed for each genotype and condition. Error bars represent standard error values. Asterisks indicate significant differences between the means of dark (black bar) and red light irradiated (red bar) samples as determined by Student's t test: *: $p < 0.05$, **: $p < 0.01$, ns: not significant.

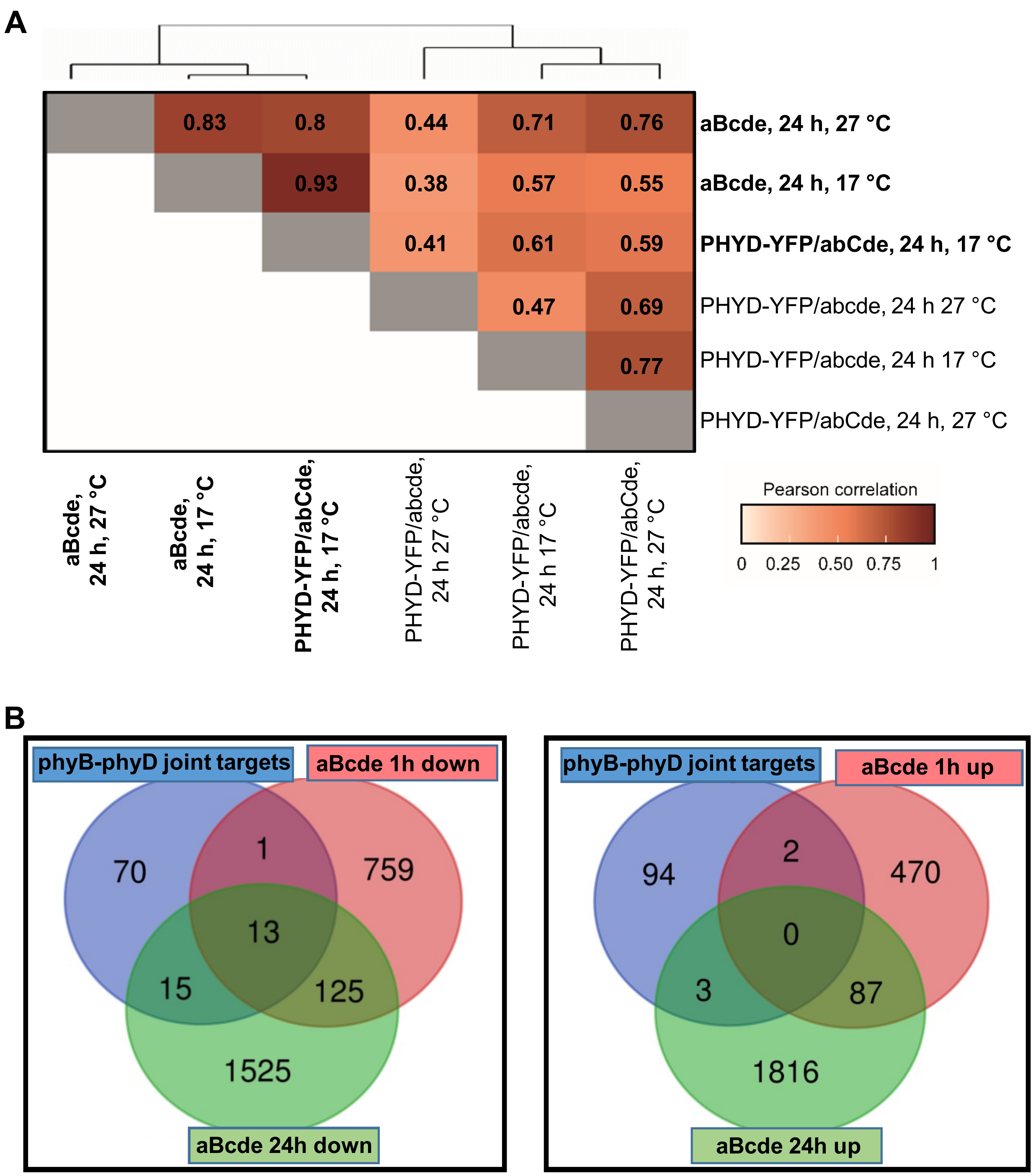

Supplementary Fig. S10. 24h Red light irradiation induced transcriptional changes of the phyB-phyD joint target genes in different Arabidopsis phytochrome mutants.

(A) Pearson correlation of expression changes of the phyB-phyD joint target genes in response to 24 h red light treatment. Plants that showed the photomorphogenic response of hypocotyl shortening under red light at the given condition are printed in bold. (B) Differentially expressed genes (DEGs) of phyB-phyD joint targets. Venn-diagram shows the overlap of phyB-phyD target genes and the DEGs of aBcde plants grown at 17 °C and treated with red light for 1 or 24 h. DEGs are the genes that showed significant and at least 2x different expression in the light treated plant relative to the dark control. Upregulated (up) and downregulated (down) genes are shown separately. Note that two genes showed mixed regulation, they were upregulated at one time point and downregulated at the other time point. Thus 32 genes out of 99 phyB-phyD targets were identified as DEGs.

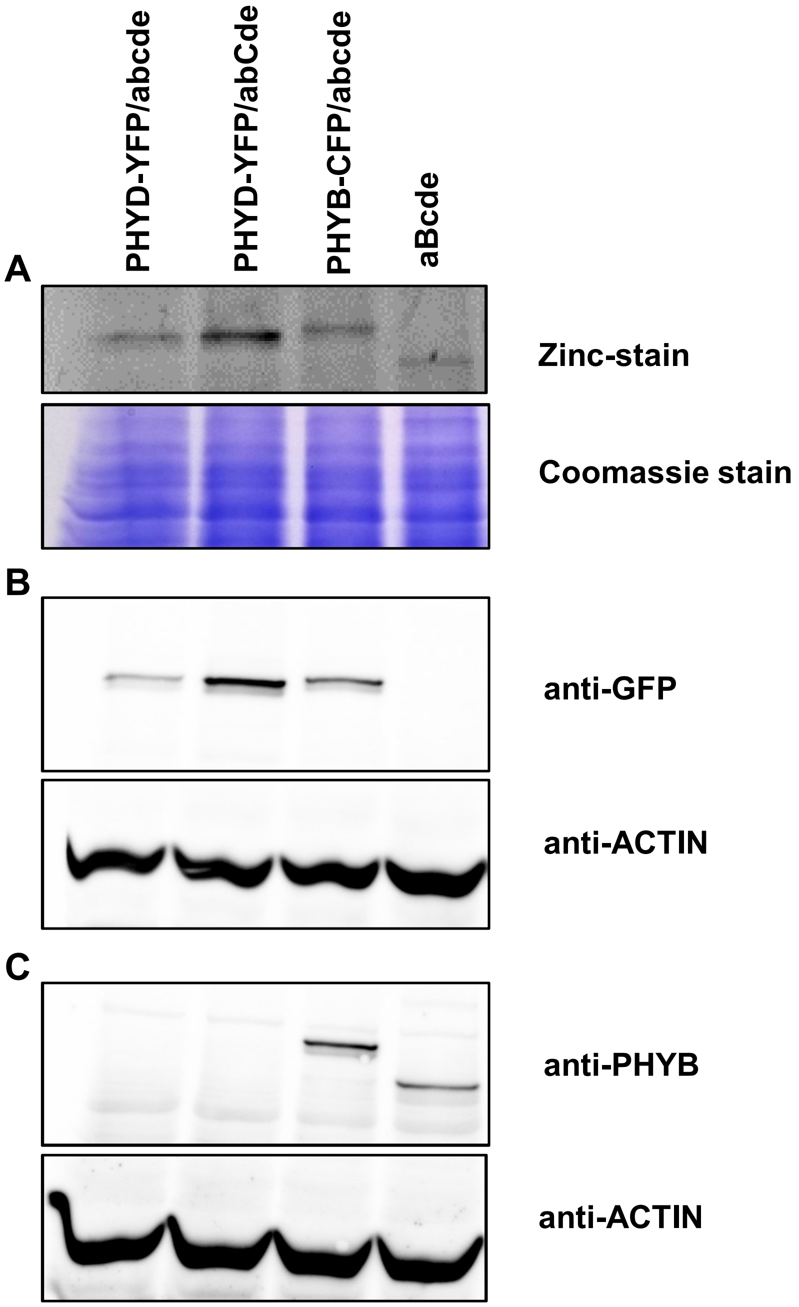

Supplementary Fig. S11. PHYD-YFP binds chromophore efficiently *in planta.* Seedlings grown on filter paper for 4 days in the dark at 17 °C and protein extracts were analyzed by (A) Zinc blot staining (Coomassie stain for loading control) and by western blot hybridization using the (B) anti-GFP or (C) anti-PHYB antibodies. ACTIN was used as loading control.

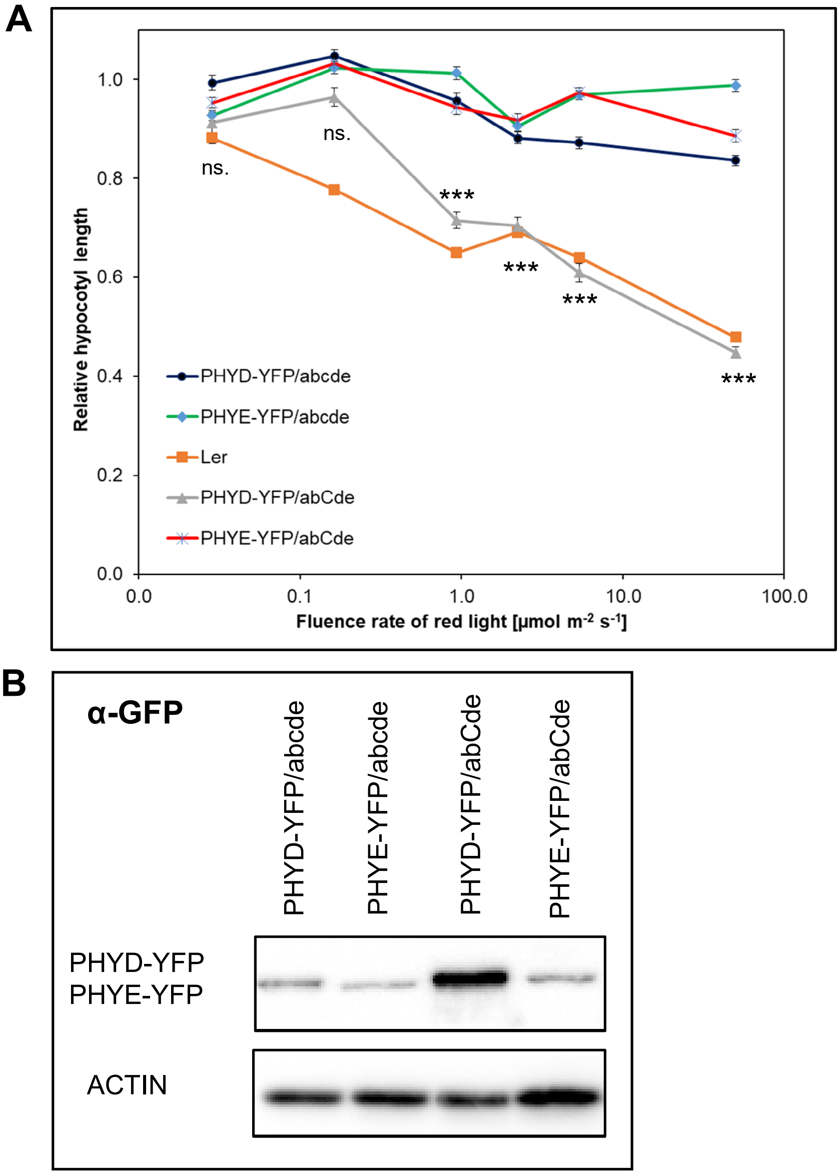

Supplementary Fig. S12. PhyC increases the accumulation and promotes the function of phyD-YFP but not that of phyE-YFP.
(A) Seedlings were grown at 17 °C for 4 days under different fluences of constant red light irradiation. Hypocotyl values relative to the corresponding dark controls are shown. n≥30, error bars indicate standard errors. Asterisks denote significant difference between the abCde lines expressing phyD-YFP or phyE-YFP at the marked data points (Mann-Whitney U test, *** $p<0.001$; ns.: not significant). (B) Seedlings were grown under constant R irradiation (50 µmol m^{-2} s^{-1}) for 4 days at 17 °C and the amounts of PHYE-YFP and PHYD-YFP proteins were determined by immunoblotting using anti-GFP antibody. ACTIN was used as loading control.

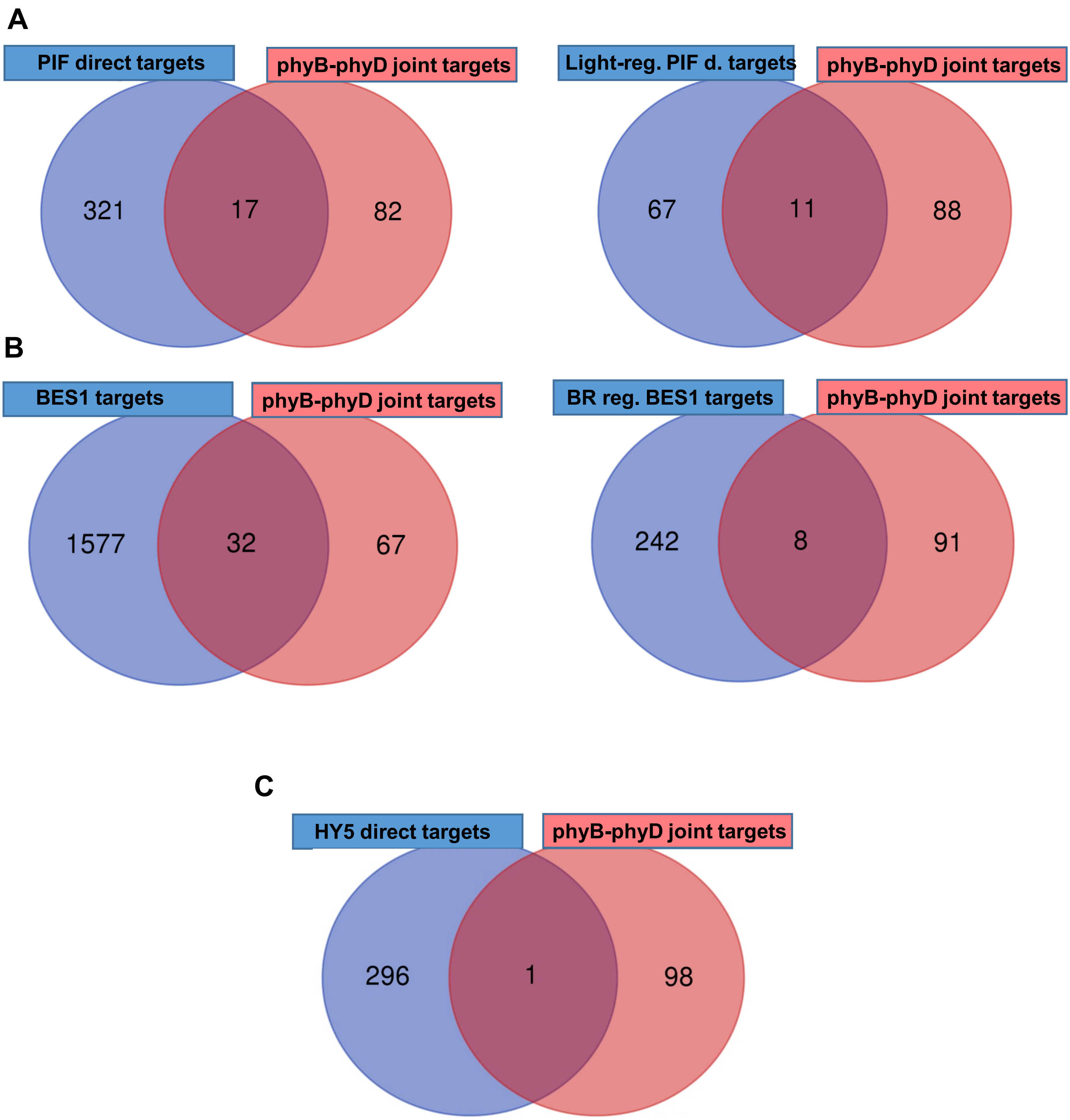

Supplementary Fig. S13. Representation of phyB-phyD joint targets among the direct targets of critical transcriptional regulators of photomorphogenesis.
(A-B) Representation of phyB-phyD joint target genes among the target lists of negative regulators of photomorphogenesis. Significance of the overlaps were tested by Fisher's Exact Test (Two-Sided) on 2x2 Matrix. The calculations were based on hypergeometric distribution. (A) Venn-diagrams show the overlap between the PIF direct targets and the light-regulated PIF direct targets (Light-reg. PIF d. targets) and the phyB-phyD joint targets (p = 1.39E-12 and p = 2.15E-13, respectively). (B) Venn-diagram shows the overlap between the BES1 targets and the brassinosteroid-regulated BES1 targets (BR reg. BES1 targets) and the phyB-phyD targets (p = 8.63E-12 and p = 4.40E-05, respectively). (C) Venn-diagram shows the overlap between the HY5 direct targets and the phyB-phyD targets (p = 1, the overlap is not significant).

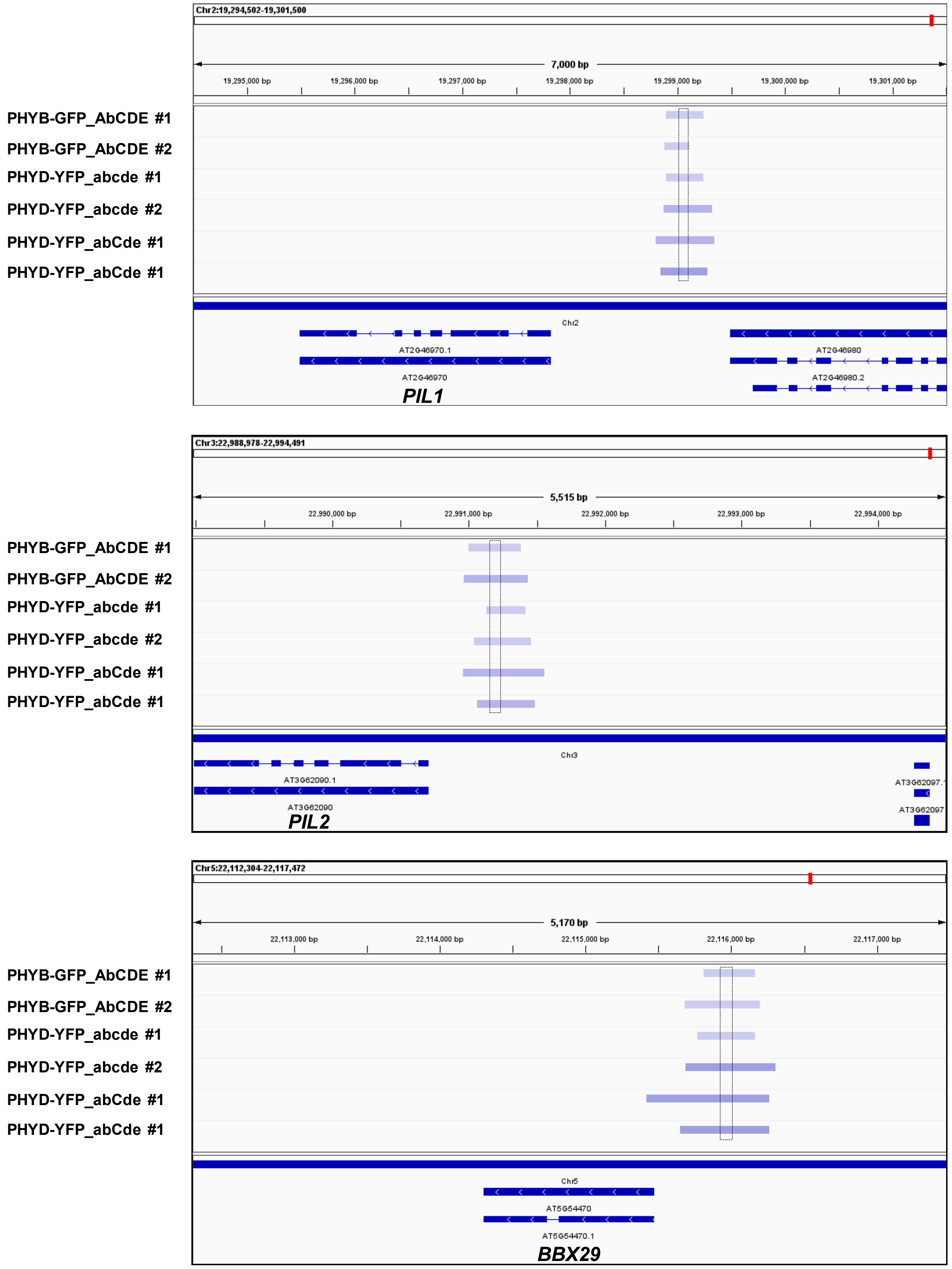

Supplementary Fig. S14. Mapping ChIP-seq sequence reads of chosen genes.
The distribution of sequence reads mapped around the genomic loci of *PIL1*, *PIL2* and *BBX29* genes. The bottom section of each box shows the gene models (Araport11) in the given genomic region. Chromosomal positions of peaks identified in different samples are represented by light blue boxes in the upper section. The corresponding samples are indicated on the left side. The vertical rectangles with dashed outline show the position of DNA fragments that were amplified and detected in ChIP-qPCR assays.
